# Hypertension awareness, treatment, and control and their association with healthcare access in the middle-aged and older Indian population: A nationwide cohort study

**DOI:** 10.1371/journal.pmed.1003855

**Published:** 2022-01-04

**Authors:** Jinkook Lee, Jenny Wilkens, Erik Meijer, T. V. Sekher, David E. Bloom, Peifeng Hu

**Affiliations:** 1 Center for Economic and Social Research, University of Southern California, Los Angeles, California, United States of America; 2 Department of Economics, University of Southern California, Los Angeles, California, United States of America; 3 International Institute for Population Sciences, Mumbai, India; 4 Harvard T.H. Chan School of Public Health, Boston, Massachusetts, United States of America; 5 Division of Geriatric Medicine, David Geffen School of Medicine, University of California, Los Angeles, Los Angeles, California, United States of America; Harvard Medical School, UNITED STATES

## Abstract

**Background:**

Hypertension is the most important cardiovascular risk factor in India, and representative studies of middle-aged and older Indian adults have been lacking. Our objectives were to estimate the proportions of hypertensive adults who had been diagnosed, took antihypertensive medication, and achieved control in the middle-aged and older Indian population and to investigate the association between access to healthcare and hypertension management.

**Methods and findings:**

We designed a nationally representative cohort study of the middle-aged and older Indian population, the Longitudinal Aging Study in India (LASI), and analyzed data from the 2017–2019 baseline wave (*N =* 72,262) and the 2010 pilot wave (*N =* 1,683). Hypertension was defined as self-reported physician diagnosis or elevated blood pressure (BP) on measurement, defined as systolic BP ≥ 140 mm Hg or diastolic BP ≥ 90 mm Hg. Among hypertensive individuals, awareness, treatment, and control were defined based on self-reports of having been diagnosed, taking antihypertensive medication, and not having elevated BP, respectively. The estimated prevalence of hypertension for the Indian population aged 45 years and older was 45.9% (95% CI 45.4%–46.5%). Among hypertensive individuals, 55.7% (95% CI 54.9%–56.5%) had been diagnosed, 38.9% (95% CI 38.1%–39.6%) took antihypertensive medication, and 31.7% (95% CI 31.0%–32.4%) achieved BP control. In multivariable logistic regression models, access to public healthcare was a key predictor of hypertension treatment (odds ratio [OR] = 1.35, 95% CI 1.14–1.60, *p* = 0.001), especially in the most economically disadvantaged group (OR of the interaction for middle economic status = 0.76, 95% CI 0.61–0.94, *p* = 0.013; OR of the interaction for high economic status = 0.84, 95% CI 0.68–1.05, *p =* 0.124). Having health insurance was not associated with improved hypertension awareness among those with low economic status (OR = 0.96, 95% CI 0.86–1.07, *p* = 0.437) and those with middle economic status (OR of the interaction = 1.15, 95% CI 1.00–1.33, *p* = 0.051), but it was among those with high economic status (OR of the interaction = 1.28, 95% CI 1.10–1.48, *p* = 0.001). Comparing hypertension awareness, treatment, and control rates in the 4 pilot states, we found statistically significant (*p <* 0.001) improvement in hypertension management from 2010 to 2017–2019. The limitations of this study include the pilot sample being relatively small and that it recruited from only 4 states.

**Conclusions:**

Although considerable variations in hypertension diagnosis, treatment, and control exist across different sociodemographic groups and geographic areas, reducing uncontrolled hypertension remains a public health priority in India. Access to healthcare is closely tied to both hypertension diagnosis and treatment.

## Abstract 2

Jinkook Lee and colleagues investigate hypertension management and its association with healthcare access in middle-aged and older adults in India.

## Author summary

**Why was this study done?:**

- Hypertension is one of the most important drivers of the rising mortality and disability associated with cardiovascular diseases in India.
- Current knowledge about hypertension management among middle-aged and older adults in India is limited.
- Striking disparities in access to healthcare exist in India, and access to healthcare might be tied to hypertension awareness and treatment.

**What did the researchers do and find?:**

- We developed a nationally representative cohort study, the Longitudinal Aging Study in India (*N =* 72,262), and investigated hypertension prevalence, awareness, treatment, and control based on both self-report of diagnosis and blood pressure measurement.
- Among hypertensive adults aged 45 years and older, 55.7% had been told by a physician that they had hypertension, 38.9% took antihypertensive medication, and 31.7% kept their blood pressure controlled, and large variations in hypertension care were found across states and sociodemographic groups.
- Access to healthcare was a key predictor of hypertension awareness and treatment, and access to a public health center was especially critical for individuals in the most economically disadvantaged group to get access to treatment.

**What do these findings mean?:**

- Striking disparities in hypertension awareness, treatment, and control exist in India, and access to healthcare is tied to these disparities.
- Hypertension management has improved much in India from 2010 to 2017–2019, and health insurance expansion and growth in public healthcare facilities are important contributors to this improvement.
- Comparisons between 2010 and 2017–2019 were based on data from only 4 states, and therefore their generalizability to other states is limited.

## Introduction

Elevated blood pressure (BP), once a public health challenge largely affecting high-income countries, is now most prevalent in low- and middle-income countries [1]. India is home to 199 million adults with elevated BP [1], and hypertension is one of the most important drivers of the rising mortality and disability associated with cardiovascular diseases [2–4]. This growing impact of hypertension has drawn substantial research attention, generating many single-center studies and a few multicenter studies in the past 6 decades [5]. Anchala and colleagues [6] conducted a meta-analysis of these studies from 1950 to 2013, which was used as the scientific basis for the 2019 Indian Guidelines on Hypertension–IV [5,7], although the studies included in this meta-analysis generally represented the Indian population poorly [4].

Since the publication of the meta-analysis, a few national-level studies on hypertension prevalence have offered important new evidence [3,8,9], but attention to hypertension management and BP control among hypertensive patients has been more limited. An exception is Prenissl and colleagues’ recent national-level study of hypertension management [9], using the data from the 2015–2016 National Family Health Survey (NFHS-4). As the NFHS-4 sample represented those aged 15 to 49 years, the authors could not investigate BP control among the population aged 50 years and older. This age exclusion was a major shortcoming, considering that middle-aged and older adults have higher hypertension prevalence. To fill this knowledge gap, the first objective of this study was to investigate hypertension awareness, treatment, and control in the middle-aged and older Indian population, using newly available, nationally representative data from the Longitudinal Aging Study in India (LASI), which represents the population aged 45 years and older.

We paid particular attention to access to healthcare, examining the association between access to healthcare and hypertension management. Access to healthcare in India has been under the spotlight, especially after the 2016 Global Burden of Disease study ranked India 145th out of 195 countries in terms of healthcare access and quality [10]. Although considerable advances have been made in the past decade, access to healthcare in India is worse than in many other middle-income countries, including India’s neighboring countries [10], and striking disparities exist across geographic locations within the country [11,12]. We hypothesized that access to healthcare is closely tied to hypertension awareness and treatment, leading to good BP control, because antihypertensive medications are both inexpensive and generally efficacious for BP control [13–16]. We used the hypertension cascade approach [9], which depicts where in the care process patients are lost to care, and examined how access to healthcare, particularly having health insurance and access to public healthcare facilities, was associated with hypertension awareness, treatment, and control. Noting the disparities across geographic locations, we examined urban/rural and cross-state differences in hypertension management, using the 2017–2019 LASI [17], which provides state-representative data from 35 states and union territories.

We then investigated the changes in hypertension management over time, using the 2010 LASI pilot data [18]; the pilot wave collected data on hypertension management using an identical protocol, from the 4 states of Karnataka, Kerala, Punjab, and Rajasthan. Pooling data from these 4 states, we examined changes in hypertension management over time in subpopulations, considering disparities between urban and rural populations and across socioeconomic groups [11,12].

## Methods

### Study design and data

We designed LASI as a nationally representative longitudinal study to provide detailed, comprehensive longitudinal data on the key health, economic, and social characteristics of India’s older adults. Using the 2011 census as the sampling frame, LASI represents the nation as a whole, as well as each state and union territory, with interviews of adults aged 45 years and older and their spouses of all ages. The prospective protocol of the project can be found in S1 Appendix. For the current analysis, we used the 2017–2019 baseline wave (*N =* 72,262) and the 2010 pilot wave (*N =* 1,683). Although much smaller in sample size, the 2010 pilot sample was recruited from 4 states, Karnataka, Kerala, Punjab, and Rajasthan, to represent the diversity of the country. Sampling weights were constructed and used in all descriptive analyses. The details of the study design have been described elsewhere [17,18]. The response rates for the 2017–2019 and 2010 waves were 87.3% and 90.9%, respectively.

For this analysis, we excluded participants younger than age 45 years (*N =* 6,687 in 2017–2019; *N =* 225 in 2010), those who did not have at least 1 valid systolic BP (SBP) and diastolic BP (DBP) measurement (*N =* 5,962 in 2017–2019; *N =* 169 in 2010), and those missing information on education, household consumption, or health insurance (*N =* 129 for 2017–2019; *N =* 3 for 2010). For 2017–2019, we also excluded those with missing information on availability of healthcare facilities in the community (*N =* 51). After these exclusions, the sample sizes were 59,433 for the 2017–2019 baseline wave and 1,286 for the 2010 pilot wave.

Data were collected via in-home interviews. Relevant to the current analysis, interviewers asked each study participant about age, sex, education, and health insurance. In the analyses, we divided age groups into 45–49 years, 50–54 years, 55–59 years, 60–64 years, 65–69 years, 70–74 years, and 75 years and older. We also conducted the analyses using 10-year age groups and found qualitatively consistent results, suggesting the same direction of age gradients and statistical significance. Education was categorized as no education, completed primary school, and secondary school or more. The binary variable indicating health insurance coverage was based on the question “Are you covered by health insurance?”

The community survey identified access to public and private healthcare facilities. This survey was administered to village/neighborhood leaders and government officials, who listed all available public and private healthcare facilities in a rural village or in an urban ward where the respondents resided. Public healthcare facilities, run mainly by state governments, provide free or very low cost medical services, which would be particularly beneficial for the poorest subpopulation. An extensive private healthcare sector also covers the entire spectrum from outpatient visits to general and specialized hospitals.

Finally, the economic status of households was measured through per capita consumption, which is considered a better measure than income or wealth in developing countries [19]. Interviewers asked the individual most knowledgeable about household finances a detailed set of household consumption questions. Missing values for consumption were imputed (see Lee and colleagues [20] for details). Per capita total household consumption was constructed by summing the itemized responses and dividing them by household size. We then categorized these values into tertiles.

### BP measurement and definition of hypertension

The 2017–2019 baseline and the 2010 pilot waves used an identical protocol to measure SBP and DBP levels [17,21]. Trained interviewers measured BP levels 3 times using an Omron automatic BP monitor. We used the mean of the second and third readings. If fewer than 3 readings were available, we used the mean of 2 readings or a single reading if only 1 reading was available. Hypertension was defined as an SBP level of 140 mm Hg or higher, a DBP level of 90 mm Hg or higher, or self-report of hypertension based on the question “Has any health professional ever told you that you have high blood pressure or hypertension?” The aforementioned BP thresholds were chosen based on the Indian guidelines on hypertension [5,7].

We defined awareness of hypertension as the self-report of diagnosed hypertension among all those classified as hypertensive. Participants who reported diagnosed hypertension were asked a follow-up question: “To control your blood pressure or hypertension, are you currently taking any medication?” Those who answered “yes” were categorized as taking antihypertensive medication (treatment). By definition, those who reported taking BP medications were all persons with hypertension. Participants who reported diagnosed hypertension were also asked about dietary restrictions: “In order to control your blood pressure, are you under salt or other diet restrictions?” Among those diagnosed, BP control was defined as an SBP level below 140 mm Hg and a DBP level below 90 mm Hg. We constructed the hypertension cascade [9] only for those with hypertension (as per the aforementioned definition), wherein the denominator was the same for each step.

### Analysis

The survey provides sampling weights that match demographic distributions within each state. We used these weights for all descriptive analyses using the 2017–2019 data. For comparisons between the 2010 and 2017–2019 data, we restricted the latter to the same 4 states as the former, and in both cases rescaled the weights such that within each state they add up to the state’s population aged 45 years and over for the closest year available from India’s Registrar General and Census Commissioner and Ministry of Health and Family Welfare (2011 census for the 2010 data [22] and 2016 projections for the 2017–2019 data [23]). India is aging rapidly, and the changes in population age structure might contribute to changes in our analytic variables, in particular BP control. Therefore, we also calculated age-standardized estimates following the World Health Organization standard population, used in conjunction with the survey weights [24].

We first estimated hypertension prevalence among adults aged 45 years and older, using data from the 2017–2019 baseline wave, and then among hypertensive individuals, we estimated hypertension awareness, treatment, and control rates. As large health disparities were previously reported across socioeconomic groups and geographic locations [11,12], we also examined the hypertension cascade by subpopulations.

We then investigated the association between access to healthcare and hypertension awareness, treatment, and control among hypertensive adults. We used unweighted multivariable logistic regression with the following covariates: age, sex, education, per capita consumption, urban/rural residence, having health insurance, and access to public and private healthcare facilities. (The regressors include the demographics that were used in constructing the weights, and therefore the main effect of weighting would be to increase variability. Thus, it is often preferred to not weight regression models [19,25].) As healthcare facilities vary considerably across states [10], we also controlled for state in our logistic regression models.

We estimated 3 separate logistic regression models for awareness, treatment, and control of hypertension. Because only those with diagnosed hypertension were asked about antihypertensive medications, we first investigated how the aforementioned factors were associated with hypertension awareness, and subsequently investigated factors associated with taking medication among those who were aware of their diagnosis. Among treated individuals, we investigated factors associated with BP control. As we hypothesized that access to public healthcare facilities would be particularly important for those with low economic status, we introduced an interaction term between access to healthcare and economic status (i.e., per capita consumption tertile). We also included an interaction between health insurance and economic status to investigate potential moderating effects. To interpret the results from the logistic regressions, we present several statistics. For some relations, we graph the predictive margins, also known as average-adjusted predictions (AAPs) [26], which are the average predicted outcomes with all covariates as observed except the one of interest, which is set to a specific value. Our main tables present the average marginal effects (AMEs), which are the differences between the AAPs of the categories of interest and the reference category, for example, the difference in average predicted awareness between men and women, controlling for the other covariates. The corresponding odds ratios (ORs) are presented in S5 Table. Because the AMEs average interaction terms out, we discuss the ORs and show AAPs for subgroups when discussing interaction effects. All analyses were conducted using Stata statistical software version 14 [27].

Finally, we studied hypertension prevalence using data from the 2017–2019 baseline wave and the 2010 pilot wave, including an investigation of mean levels of SBP and DBP. We pooled the data from the 4 pilot states, Karnataka, Kerala, Punjab, and Rajasthan, for this comparison. Among hypertensive individuals, we compared hypertension awareness, treatment, and control in the 2 time periods of 2010 and 2017–2019, and report *t*-statistics. We compared the hypertension cascades of the 2 time periods for urban and rural locations separately and by subpopulations.

This study is reported as per the REporting of studies Conducted using Observational Routinely-collected Data (RECORD) guideline (S1 RECORD Checklist).

### Ethics statement

LASI obtained approval from the University of Southern California Institutional Review Board (IRB) (UP-CG-14_00005), the Harvard University IRB (CR-16715-10), and the International Institute for Population Sciences IRB (Sr. No. 12/1054), and Health Ministry’s Screening Committee clearance from the Indian Council of Medical Research (F.No.T.21012/07/2012-NCD). Each LASI participant provided written consent for participation.

### Patient and public involvement

No patients were involved in setting the research question or the outcome measures, nor were they involved in developing plans for study design or implementation. No patients were asked to advise on the interpretation or writing up of results.

## Results

Table 1 presents the sample characteristics. The sample includes 59,433 adults aged 45 years and older in 2017–2019. The characteristics of the excluded sample can be found in S1 Table. The excluded sample includes a greater proportion of those aged 75 years and older, males, urban residents, and those with high socioeconomic status than the analysis sample (*p <* 0.01). We investigated the potential for selectivity bias in our estimates due to nonresponse by constructing a new set of weights for our baseline wave analysis sample: The results were very similar, usually within 1 percentage point, suggesting no evidence of selectivity bias (S2 Table). We also investigated the potential bias associated with imputation of missing values for per capita consumption by comparing distributions for the sample including the imputed cases with distributions for the sample that excludes them (S3 Table). We found no meaningful differences, and therefore conclude that there is no indication of selection bias. The estimated prevalence of hypertension weighted to the Indian population was 45.9% (95% CI 45.4%–46.5%) in 2017–2019. Table 1 also shows hypertension prevalence estimates by sociodemographic characteristics.

**Table 1:** Sample characteristics and hypertension prevalence rates.

Characteristic	Sample *N* (%)	Hypertension prevalence rate	
		*N* (%)	95% CI
All	59,433 (100%)	29,288 (45.9)	45.4, 46.5
Age (years)			
45–49	11,985 (20.2)	4,399 (34.6)	33.4, 35.8
50–54	9,934 (19.2)	4,333 (40.7)	39.5, 41.9
55–59	9,118 (17.7)	4,377 (44.8)	43.6, 46.1
60–64	9,269 (14.1)	4,896 (49.9)	48.7, 51.2
65–69	8,056 (12.7)	4,586 (54.2)	52.8, 55.5
70–74	5,192 (7.5)	3,107 (56.5)	54.8, 58.1
75+	5,879 (8.5)	3,590 (58.6)	57.1, 60.2
Sex			
Male	27,518 (53.2)	13,050 (42.6)	41.8, 43.3
Female	31,915 (46.8)	16,238 (49.8)	49.1, 50.5
Urbanicity			
Rural	38,868 (68.6)	17,593 (41.8)	41.2, 42.4
Urban	20,565 (31.4)	11,695 (54.9)	54.0, 55.8
Education			
None	27,980 (52.4)	13,071 (43.6)	42.9, 44.3
Primary school	14,830 (21.8)	7,498 (47.5)	46.5, 48.6
Secondary school	16,623 (25.8)	8,719 (49.4)	48.4, 50.4
Per capita consumption			
First tertile	20,166 (36.9)	8,889 (40.7)	39.8, 41.5
Second tertile	19,792 (33.3)	9,862 (46.6)	45.8, 47.5
Third tertile	19,475 (29.8)	10,537 (51.7)	50.8, 52.6
Has health insurance			
No	45,680 (78.5)	22,738 (45.8)	45.2, 46.4
Yes	13,753 (21.5)	6,550 (46.4)	45.4, 47.5
Access to public health center			
No	45,679 (78.3)	21,997 (44.8)	44.2, 45.3
Yes	13,754 (21.7)	7,291 (50.2)	49.2, 51.3

*N*s are unweighted; percentages use national weights.

We report hypertension awareness, treatment, and control among hypertensive individuals in Table 2. For the hypertensive participants included in this analysis, the mean age was 61.9 years in 2017–2019 (*N =* 29,288). Table 2 depicts the age-standardized proportion of hypertensive adults who reached each step of the care cascade. In 2017–2019, 55.7% (95% CI 54.9%–56.5%) had been diagnosed (“awareness”), 38.9% (95% CI 38.1%–39.6%) reported currently taking antihypertensive medication (“treatment”), and 31.7% (95% CI 31.0%–32.4%) had a normal BP (“control”). We observe sharp age gradients in hypertension awareness (Σ^2^ = 178.5, *p <* 0.001) and treatment (Σ^2^ = 512.1, *p <* 0.001), but much reduced gradients in BP control (Σ^2^ = 15.6, *p =* 0.016). A greater proportion of females and urban residents reached each step of the care cascade compared with males and rural residents, all at the significance level *p* < 0.001. Hypertension awareness and treatment rates were about 7 to 8 percentage points higher among adults with secondary school or more education compared with those with no education (awareness: 53.7% [95% CI 52.5%–54.9%] for those with no education and 60.4% [95% CI 59.1%–61.7%] for those with secondary school or more; treatment: 34.5% [95% CI 33.4%–35.6%] for those with no education and 46.4% [95% CI 45.1%–47.7%] for those with secondary school or more). Hypertension awareness, treatment, and control rates varied across per capita consumption tertiles, ranging from 48.6% (95% CI 47.1%–50.0%) to 62.0% (95% CI 60.7%–63.2%) for awareness, 29.2% (95% CI 28.0%–30.5%) to 47.3% (95% CI 46.0%–48.5%) for treatment, and 28.3% (95% CI 27.0%–29.6%) to 36.0% (95% CI 34.8%–37.2%) for control.

**Table 2:** Cascade of hypertension care.

Characteristic	Awareness		Treatment		Control	
	*N* (%) or Σ^2^	95% CI or *p*-value	*N* (%) or Σ^2^	95% CI or *p*-value	*N* (%) or Σ^2^	95% CI or *p*-value
All	17,034 (55.7)	54.9, 56.5	12,307 (38.9)	38.1, 39.6	9,255 (31.7)	31.0, 32.4
Age (years)						
45–49	2,265 (48.0)	45.9, 50.1	1,351 (28.4)	26.6, 30.2	1,347 (29.0)	27.2, 30.9
50–54	2,380 (54.4)	52.5, 56.2	1,539 (34.3)	32.5, 36.1	1,359 (32.7)	30.9, 34.5
55–59	2,516 (56.0)	54.1, 57.8	1,790 (39.1)	37.3, 40.9	1,402 (31.8)	30.1, 33.6
60–64	2,850 (57.8)	56.1, 59.5	2,155 (43.3)	41.6, 45.0	1,558 (32.8)	31.2, 34.5
65–69	2,878 (61.1)	59.3, 62.9	2,206 (46.6)	44.8, 48.3	1,514 (32.7)	31.1, 34.4
70–74	1,936 (60.7)	58.6, 62.9	1,534 (48.1)	45.9, 50.3	1,015 (33.6)	31.6, 35.7
75+	2,209 (61.2)	59.2, 63.2	1,732 (47.7)	45.7, 49.8	1,060 (31.1)	29.2, 33.0
Σ^2^, *p-*value	178.5	<0.001	512.1	<0.001	15.6	0.016
Sex						
Male	6,725 (49.7)	48.6, 50.8	4,788 (34.3)	33.3, 35.4	3,511 (27.2)	26.2, 28.2
Female	10,309 (62.6)	61.7, 63.6	7,519 (43.8)	42.8, 44.7	5,744 (37.3)	36.3, 38.3
Σ^2^, *p-*value	424.9	<0.001	274.6	<0.001	240.1	<0.001
Urbanicity						
Rural	9,452 (52.1)	51.1, 53.1	6,204 (32.8)	31.9, 33.7	5,169 (30.5)	29.6, 31.4
Urban	7,582 (61.8)	60.6, 63.0	6,103 (49.0)	47.8, 50.2	4,086 (33.7)	32.6, 34.9
Σ^2^, *p-*value	356.0	<0.001	825.6	<0.001	100.4	<0.001
Education						
None	7,228 (53.7)	52.5, 54.9	4,897 (34.5)	33.4, 35.6	3,983 (31.8)	30.7, 32.9
Primary school	4,461 (56.3)	54.8, 57.8	3,297 (41.2)	39.7, 42.6	2,400 (31.9)	30.5, 33.3
Secondary school	5,345 (60.4)	59.1, 61.7	4,113 (46.4)	45.1, 47.7	2,872 (32.6)	31.3, 33.9
Σ^2^, *p-*value	84.9	<0.001	218.1	<0.001	15.5	<0.001
Per capita consumption						
First tertile	4,372 (48.6)	47.1, 50.0	2,769 (29.2)	28.0, 30.5	2,349 (28.3)	27.0, 29.6
Second tertile	5,816 (56.3)	55.0, 57.7	4,222 (39.8)	38.5, 41.0	3,080 (30.6)	29.4, 31.8
Third tertile	6,846 (62.0)	60.7, 63.2	5,316 (47.3)	46.0, 48.5	3,826 (36.0)	34.8, 37.2
Σ^2^, *p-*value	497.8	<0.001	741.0	<0.001	218.9	<0.001

*N*s are unweighted; percentages use age-standardized, national weights.

**Fig 1:**
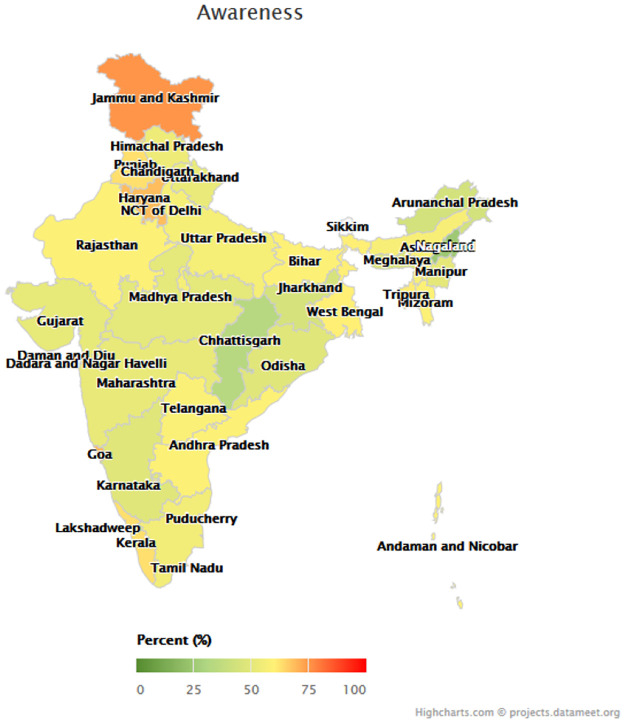
Hypertension awareness rates by state. *N =* 29,288 participants; figure uses age-standardized state weights. Colors of state names only differ to improve the readability of the map. Parliamentary constituencies map provided by Data{Meet} Community Created Maps of India (http://projects.datameet.org/maps/), made available under Creative Commons Attribution 2.5 India (http://creativecommons.org/licenses/by/2.5/in/).

**Fig 2:**
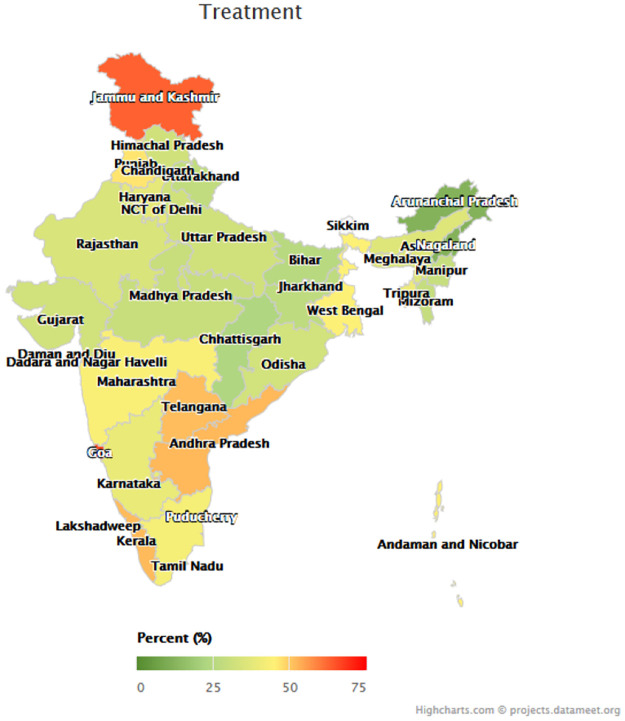
Hypertension treatment rates by state. *N =* 29,288 participants; figure uses age-standardized state weights. Colors of state names only differ to improve the readability of the map. Parliamentary constituencies map provided by Data{Meet} Community Created Maps of India (http://projects.datameet.org/maps/), made available under Creative Commons Attribution 2.5 India (http://creativecommons.org/licenses/by/2.5/in/).

**Fig 3:**
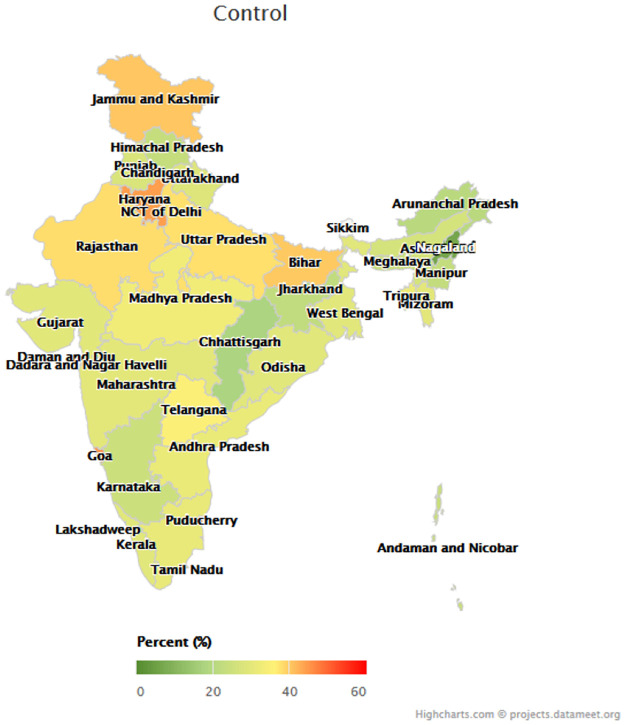
Hypertension control rates by state. *N =* 29,288 participants; figure uses age-standardized state weights. Colors of state names only differ to improve the readability of the map. Parliamentary constituencies maps provided by Data{Meet} Community Created Maps of India (http://projects.datameet.org/maps/), made available under Creative Commons Attribution 2.5 India (http://creativecommons.org/licenses/by/2.5/in/).

Figs 1–3 depict the variation among states in the proportion of middle-aged and older adults with hypertension who reached each step of the care cascade. Awareness of diagnosis (Fig 1) ranged from 24.8% (95% CI 21.1%–28.8%) in Nagaland and 33.3% (95% CI 30.1%–36.6%) in Chhattisgarh to 69.6% (95% CI 65.2%–73.8%) in Goa and 75.2% (95% CI 71.1%–78.9%) in Jammu and Kashmir. Treatment rates (Fig 2) ranged from 11.9% (95% CI 9.5%–14.9%) in Nagaland and 12.0% (95% CI 9.1%–15.7%) in Arunachal Pradesh to 65.4% (95% CI 61.0%–69.6%) in Goa, and control rates (Fig 3) varied from 6.4% (95% CI 4.6%–8.8%) in Nagaland to 45.4% (95% CI 41.0%–49.8%) in Goa. S4 Table presents the age-standardized hypertension awareness, treatment, and control rates in each state/union territory.

Using data from the 2017–2019 wave, we investigated the association between access to healthcare and hypertension management among hypertensive adults in multivariable logistic regression models. We estimated 3 logistic regression models, first estimating awareness among all hypertensive adults (*N =* 29,288). Then, among those diagnosed with hypertension, we examined the association between access to healthcare and treatment (*N =* 17,034). Finally, among the hypertensive adults on antihypertensive medication, we investigated the factors associated with BP control (*N =* 12,307). Table 3 presents AMEs from the 3 logistic regression models, and ORs are presented in S5 Table. We also examined nonpharmacological treatment (i.e., less salt or other dietary restriction to control BP) and present the findings in S6 Table.

**Table 3:** Average marginal effects (AMEs) from the multivariable logistic regression analyses.

Characteristic	Hypertension awareness, among hypertensive adults, *N* = 29,288			Taking medication, among adults diagnosed with hypertension, *N* = 17,034			BP control, among hypertensive adults taking medication, *N* = 12,307		
	AME	95% CI	*p*-Value	AME	95% CI	*p*-Value	AME	95% CI	*p*-Value
Age (years)									
45–49	0 (Reference)			0 (Reference)			0 (Reference)		
50–54	0.040	0.019, 0.060	<0.001	0.044	0.018, 0.070	0.001	−0.007	−0.043, 0.029	0.696
55–59	0.068	0.048, 0.088	<0.001	0.113	0.087, 0.138	<0.001	−0.006	−0.041, 0.029	0.744
60–64	0.082	0.062, 0.102	<0.001	0.159	0.135, 0.183	<0.001	−0.020	−0.054, 0.013	0.238
65–69	0.129	0.109, 0.149	<0.001	0.161	0.137, 0.185	<0.001	−0.025	−0.058, 0.009	0.147
70–74	0.131	0.109, 0.153	<0.001	0.193	0.167, 0.219	<0.001	−0.024	−0.061, 0.012	0.188
75+	0.130	0.109, 0.151	<0.001	0.192	0.167, 0.217	<0.001	−0.071	−0.107, −0.036	<0.001
Sex									
Male	0 (Reference)			0 (Reference)			0 (Reference)		
Female	0.143	0.131, 0.154	<0.001	0.047	0.033, 0.061	<0.001	0.021	0.001, 0.040	0.035
Urbanicity									
Rural	0 (Reference)			0 (Reference)			0 (Reference)		
Urban	0.073	0.060, 0.086	<0.001	0.089	0.074, 0.104	<0.001	0.002	−0.018, 0.023	0.825
Education									
None	0 (Reference)			0 (Reference)			0 (Reference)		
Primary school	0.067	0.053, 0.081	<0.001	0.038	0.021, 0.055	<0.001	0.019	−0.004, 0.043	0.102
Secondary school	0.076	0.061, 0.091	<0.001	0.070	0.052, 0.087	<0.001	0.021	−0.003, 0.045	0.093
Per capita consumption									
First tertile	0 (Reference)			0 (Reference)			0 (Reference)		
Second tertile	0.066	0.052, 0.080	<0.001	0.060	0.043, 0.077	<0.001	−0.002	−0.026, 0.022	0.889
Third tertile	0.110	0.095, 0.125	<0.001	0.084	0.067, 0.101	<0.001	0.032	0.008, 0.057	0.009
Has health insurance									
No	0 (Reference)			0 (Reference)			0 (Reference)		
Yes	0.020	0.005, 0.034	0.007	−0.002	−0.020, 0.015	0.801	−0.0002	−0.023, 0.023	0.987
Access to public health center									
No	0 (Reference)			0 (Reference)			0 (Reference)		
Yes	0.008	−0.006, 0.023	0.248	0.024	0.008, 0.040	0.003	−0.003	−0.025, 0.019	0.778
Access to private health facility									
No	0 (Reference)			0 (Reference)			0 (Reference)		
Yes	−0.007	−0.020, 0.006	0.291	0.002	−0.013, 0.017	0.786	−0.011	−0.032, 0.010	0.313

Unweighted regression analyses; analyses controlled for state (AMEs not listed).

**Fig 4:**
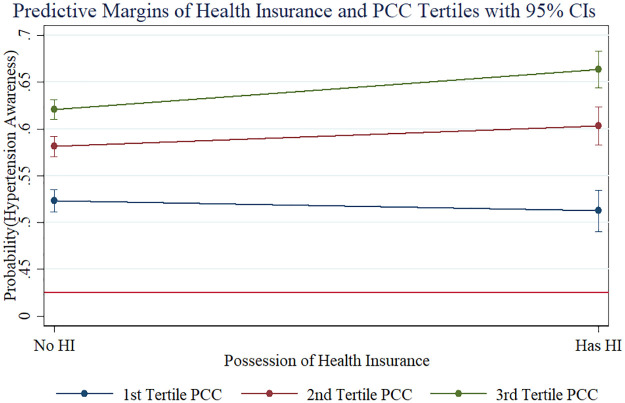
Interaction effects of economic status and health insurance on hypertension awareness. Note: HI (Health Insurance), PCC (Per Capita Consumption).

As Table 3 shows, hypertension awareness was positively associated with older age, being female, residing in an urban area, higher education, and more economic resources: For example, those aged 65–69 years were about 12.9 percentage points (95% CI 10.9–14.9, *p <* 0.001) more likely to be aware than those aged 45–49 years after controlling for all other covariates. After controlling for all other covariates, being female increased the probability of being aware by 14.3 percentage points (95% CI 13.1–15.4, *p <* 0.001), and living in an urban area rather than a rural area increased the probability by 7.3 percentage points (95% CI 6.0–8.6, *p <* 0.001). Compared with hypertensive adults with no education, primary education increased the probability of being aware by 6.7 percentage points (95% CI 5.3–8.1, *p <* 0.001), and secondary school education or more increased the probability by 7.6 percentage points (95% CI 6.1–9.1, *p <* 0.001). Compared with those with the lowest economic resources, those in the richest tertile were 11.0 percentage points (95% CI 9.5–12.5, *p <* 0.001) more likely to be aware, and those in the middle economic group were 6.6 percentage points (95% CI 5.2–8.0, *p <* 0.001) more likely to be aware. Hypertension awareness was also positively associated with having health insurance (AME = 0.020, 95% CI 0.005–0.034, *p =* 0.007). However, the latter obscures an interaction effect between having health insurance and economic status on hypertension awareness, as shown in the ORs in S5 Table: Health insurance was not associated with hypertension awareness in the poorest tertile (OR = 0.96, 95% CI 0.86–1.07, *p =* 0.437), but the interaction effect of the richest tertile was highly statistically significant (OR = 1.28, 95% CI 1.10–1.48, *p =* 0.001). For the middle category, there was weak evidence of an interaction effect (OR = 1.15, 95% CI 1.00–1.33, *p =* 0.051). Fig 4 illustrates these interaction effects.

**Fig 5:**
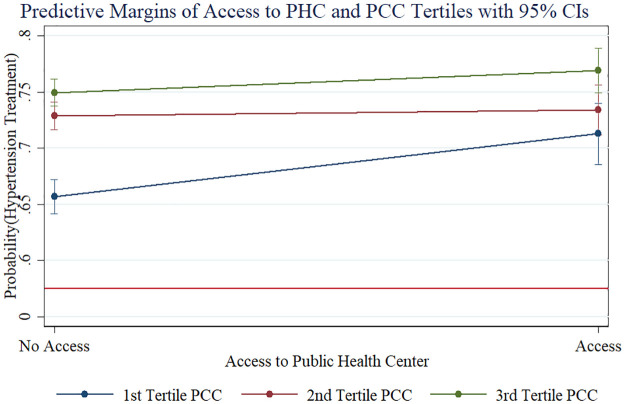
Interaction effects of economic status and access to a public health center on hypertension treatment. Note: PHC (Public Health Center), PCC (Per Capita Consumption).

Among the hypertensive individuals who were aware of their hypertension, hypertension treatment was positively associated with older age, being female, residing in an urban area, higher education, and more economic resources: Compared with individuals aged 45–49 years, individuals aged 65–69 years were about 16.1 percentage points (95% CI 13.7–18.5, *p <* 0.001) more likely to take antihypertensive medication after controlling for all other covariates, and individuals aged 70–74 years were about 19.3 percentage points (95% CI 16.7–21.9, *p <* 0.001) more likely to take medication. After controlling for all other covariates, being female increased the probability of taking medication by 4.7 percentage points (95% CI 3.3–6.1, *p <* 0.001), and living in an urban area rather than a rural area increased the probability by 8.9 percentage points (95% CI 7.4–10.4, *p <* 0.001). Compared with hypertensive adults with no education, primary education increased the probability of taking medication by 3.8 percentage points (95% CI 2.1–5.5, *p <* 0.001), and secondary school education or more increased the probability by 7.0 percentage points (95% CI 5.2–8.7, *p <* 0.001). Compared with those with the lowest economic resources, those in the richest tertile were 8.4 percentage points (95% CI 6.7–10.1, *p <* 0.001) more likely to take medication, and those in the middle economic group were 6.0 percentage points (95% CI 4.3–7.7, *p <* 0.001) more likely to take medication. As hypothesized, we found a statistically significant effect of access to a public health center on hypertension treatment (AME = 0.024, 95% CI 0.008–0.040, *p =* 0.003). Again, there was an interaction of this effect with economic status: Access to a public health center mattered more for those with more limited economic resources. Fig 5 presents this interaction effect, showing the adjusted probability of hypertension treatment by access to a public health center across per capita consumption tertiles.

The results in Table 3 also show that, conditional on treatment, only age 75+ years, sex, and the third tertile of per capita consumption were statistically significantly associated with BP control at the 5% level. Specifically, BP control was negatively associated with older age (AME of age 75+ years compared to age 45–49 years = −0.071, 95% CI −0.107 to −0.036, *p <* 0.001), but positively associated with being female (AME = 0.021, 95% CI 0.001–0.040, *p =* 0.035) and being in the highest tertile of per capita consumption (AME compared to lowest tertile = 0.032, 95% CI 0.008–0.057, *p =* 0.009). Importantly, treatment effectiveness for hypertension does not vary with health insurance or access to a public or private health facility.

Finally, we studied changes in hypertension management over time using data from the 2017–2019 baseline wave and the 2010 pilot wave. We pooled the data from the 4 pilot states, Karnataka, Kerala, Punjab, and Rajasthan, for this comparison and report the age-adjusted rates. The characteristics of each state’s sample and of the pooled sample can be found in S7 and S8 Tables, respectively.

**Fig 6:**
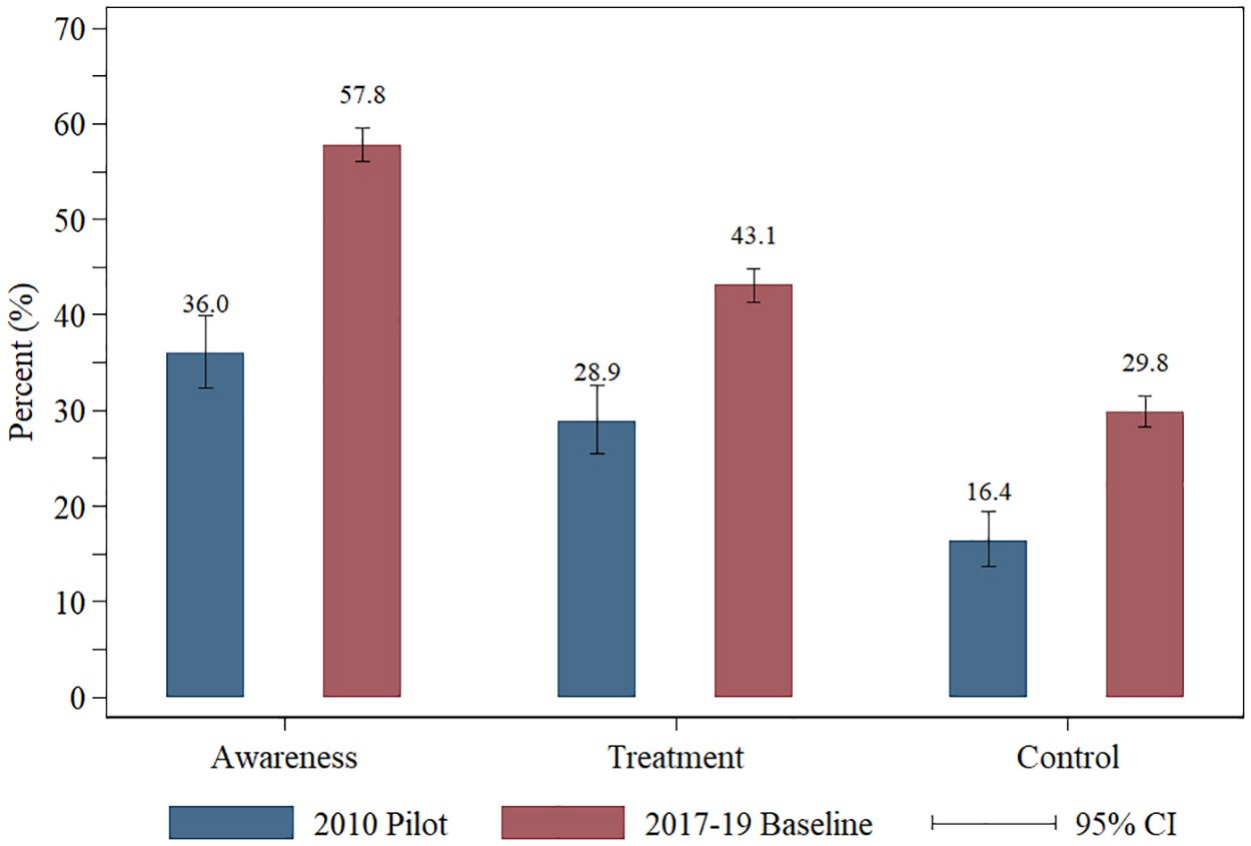
Hypertension care cascade for the 4-state pooled sample: 2010 versus 2017–2019. Hypertensive adults aged 45+ years (*N* = 645 for 2010, *N* = 4,212 for 2017–2019); the figure uses age-standardized pooled weights, and only uses the sample from Karnataka, Kerala, Punjab, Rajasthan.

Fig 6 depicts the age-standardized proportion of hypertensive adults who reached each step of the care cascade in 2010 and 2017–2019, showing large improvements over this time period. In 2010, 36.0% (95% CI 32.3%–39.9%) of the persons with hypertension had been diagnosed, 28.9% (95% CI 25.5%–32.6%) reported currently taking antihypertensive medication, and 16.4% (95% CI 13.7%–19.4%) had a normal BP. By 2017–2019, the awareness rate improved more than 20 percentage points (57.8%, 95% CI 56.1%–59.6%), and the treatment rate (i.e., the proportion taking antihypertensive medication) and the hypertension control rate improved by about 14 percentage points (treatment: 43.1%, 95% CI 41.4%–44.8%; hypertension control: 29.8%, 95% CI 28.3%–31.5%). Specifically, the changes were 21.8 (95% CI 17.6–26.0, *p <* 0.001), 14.2 (95% CI 10.4–18.0, *p <* 0.001), and 13.4 (95% CI 10.3–16.5, *p <* 0.001) percentage points for awareness, taking medication, and hypertension control, respectively. Test statistics for changes in awareness, treatment, and control were 10.26, 7.32, and 8.50, respectively, all significant at *p <* 0.001.

All subpopulations showed substantial improvements in hypertension awareness, treatment, and control over this time period, but those with lower socioeconomic status showed greater improvements. The proportion with BP control among hypertensive patients in the poorest tertile of per capita consumption increased most, from 8.7% (95% CI 5.9%–12.8%) in 2010 to 28.5% (95% CI 25.0%–32.3%) in 2017–2019, whereas the improvement in BP control rate was more modest in the richest tertile, from 28.1% (95% CI 22.5%–34.4%) to 31.2% (95% CI 28.9%–33.7%). See S8 Table for the estimates of hypertension awareness, treatment, and control for each subpopulation of the pooled sample.

The age-standardized mean BP levels among hypertensive adults also declined between 2010 and 2017–2019 (S9 Table). The mean SBP levels decreased from 147.7 mm Hg (95% CI 146.0–149.4) to 140.2 mm Hg (95% CI 139.6–140.8), and the mean DBP level decreased from 92.1 mm Hg (95% CI 91.1–93.0) to 87.5 mm Hg (95% CI 87.1–87.8). Using hypertension stages as defined by 2019 Indian guidelines [5,7], decreases occurred in all 3 hypertensive stages between the 2 waves; the reduction of stage 3 hypertension (SBP ≥ 180 mm Hg or DBP ≥ 110 mm Hg) was especially substantial, from 11.6% (95% CI 9.3%–14.4%) to 1.9% (95% CI 1.5%–2.4%).

We further examined state-level changes in awareness, treatment, and control in the 4 pilot states in 2010 and 2017–2019 and found consistent improvement except for Kerala (S10 Table), which had much higher levels of awareness, treatment, and control in 2010 than the other states. Kerala is the most advanced state in terms of epidemiological transition and has provided fairly reliable basic healthcare for all through public and private healthcare services since the 1990s [2,24]. Hence, the reason Kerala does not show large improvements in hypertension management is likely because it was already quite good in 2010.

## Discussion

### Principal findings

Data from this nationally representative sample of adults aged 45 years and older in India indicate that slightly more than half (55.7%) of hypertensive individuals had been diagnosed, less than 2 in 5 (38.9%) took medication, and less than one-third (31.7%) achieved BP control. Therefore, the highest absolute losses to care occurred at the awareness stage (45.3 percentage points) and treatment stage (16.8 percentage points). This highlights a particular need for interventions that focus on awareness and treatment. Consistent with recent findings [28], we found substantial variation in hypertension management across socioeconomic groups, sexes, and geographic areas, calling for targeting of interventions for those currently lagging behind.

We found that access to healthcare was associated with hypertension awareness and management. Having health insurance was positively associated with hypertension awareness, but this positive relationship did not hold for the poorest tertile of per capita consumption. Health insurance in India typically pays for only inpatient hospitalization and treatment at hospitals [29,30], and its impact seems to remain modest in improving hypertension diagnosis, whereas residing in a community with a public health center increased the probability of hypertensive adults taking antihypertension medication across the economic spectrum.

Moreover, access to a public health center was particularly important for those with low economic status, highlighting the importance of free or very low cost medication provided by a public health center in achieving BP control. Pooled data from 4 states (Karnataka, Kerala, Punjab, and Rajasthan) in 2010 (1,286) and 2017–2019 (*N =* 7,804) suggested large improvements in hypertension awareness, treatment, and control, except for Kerala, which already had much better hypertension management in 2010. There were secular trends in urbanicity and economic development, which might have contributed to improvements in overall hypertension care, but our subpopulation analysis further suggests that hypertension care has improved among each subpopulation across urbanicity and socioeconomic status categories. Among subpopulations, the biggest improvement was observed among those with low economic status, whereas improvement was modest among those with high economic status.

### Strengths and limitations of this study

To our knowledge, this is the largest nationally representative study of the middle-aged and older Indian population (*N =* 72,262) to date to assess hypertension prevalence, diagnosis, treatment, and control. As our sample represents adults aged 45 years and older both in the country overall and in each state and union territory, we were able to examine the hypertension care cascade at the national and state levels. The large sample size also allowed us to investigate subpopulation-level differences. Evidence showing where in the hypertension care process individuals were lost to care and how this was associated with access to healthcare provides important insights for designing appropriate interventions to improve BP control.

Study limitations include the mostly cross-sectional nature of our data. Although the 2010 pilot sample was drawn from the 2001 census to be representative of the country, its sample size was relatively small, and community-level data on access to public health facilities were not collected for the 2010 pilot study. We were only able to report changes in the hypertension care cascade comparing 4 pilot states, and therefore the observed changes may not be generalizable to other states.

### Comparison with other studies

A previous systematic review of studies from 1950 to 2013 suggested that overall estimates of BP control among hypertensive individuals aged 18 years and older were 10.7% for rural Indians and 20.2% for urban Indians [6]. Our results showed major improvements in BP control rates among both rural and urban Indians (30.5% and 33.7% in 2017–2019). Our current rate of BP control, particularly among the economically well-to-do group (36.0%), is approaching that of more developed countries. In a recent study in the United States, the estimated age-adjusted proportion of hypertensive individuals with controlled BP was 43.7% in 2017–2018 [31].

The country as a whole, however, still lags behind more developed countries. For example, Marshall and colleagues [32] reported that hypertension awareness among American and English adults aged 50 years and older was 84% and 76%, respectively, more than 20 percentage points higher than in India. However, the rates in our data resembled those of Chinese adults aged 45 years and older with hypertension, which were 57% for men and 59% for women [33].

### Research implications

Over the past decade, health insurance coverage in India has expanded substantially. In 2018, the Government of India launched the world’s largest health insurance program, Pradhan Mantri Jan Arogya Yojana (PM-JAY) (often referred to as Modicare) [34], promising free coverage for half a billion of India’s poorest citizens, which should help to continue the positive trajectory in BP control among hypertensive patients. However, we found that having health insurance was not associated with hypertension diagnosis for the poor. As noted earlier, health insurance in India typically pays for only inpatient hospitalization [29,30], and our finding supports the argument Baru et al. [35] make that even small healthcare expenses exclude the poorest individuals from utilizing healthcare. Thus, barriers to receiving care continue to exist even with health insurance, and further promotion of, and lower barriers to, preventative healthcare will need to be prioritized to reach these individuals.

Access to public health facilities was positively associated with taking antihypertensive medication. Strengthening primary healthcare is another important pillar of PM-JAY [36]. Until recently, almost all government health spending was on building public health facilities on the supply side. In 2005, the largest centrally funded supply-side program, the National Rural Health Mission, was launched, focusing on strengthening public health facilities in rural areas. A companion initiative, the National Urban Health Mission, was launched in 2014 to support urban areas [37]. Growth in public health facilities has been steady, with annual growth rates of 6.7% during 2012–2017 [38], and this expansion seems to have contributed to increased antihypertensive medication usage, leading to improved BP control.

### Conclusions

In this study, we observed that BP control has substantially improved in India during the past decade. India now has many fewer undiagnosed hypertension cases and better BP control among hypertensive patients, outcomes that are associated with increased healthcare availability. Especially encouraging is that the largest improvements in BP control occurred among those most disadvantaged in education and economic standing. The Indian government’s launch of the world’s largest health insurance program and the current challenges of the COVID-19 pandemic have sharply delineated India’s longstanding need for universal health coverage. Improving healthcare access should help to continue the positive trajectory in BP control. LASI will be able to follow its baseline wave respondents over time and evaluate how the new insurance program may increase healthcare utilization and affect hypertension awareness and management in the coming years.

## Supporting information

S1 Appendix Prospective protocol for the Longitudinal Aging Study in India. (DOCX)

S1 Code Code used to produce analytic dataset. (DOCX)

S1 RECORD Checklist (DOCX)

S1 Table Characteristics of selected and excluded sample. (XLSX)

S2 Table Sample characteristics and hypertension prevalence rates using adjusted weights. (XLSX)

S3 Table Sample characteristics and hypertension prevalence rates using non-imputed per capita consumption. (XLSX)

S4 Table Cascade of hypertension care by state. (XLSX)

S5 Table Odds ratios from multivariable logistic regression analyses for hypothesized interaction effects between economic status and access to healthcare. (XLSX)

S6 Table Average marginal effects of controlling diet among adults diagnosed with hypertension. (XLSX)

S7 Table State-specific sample characteristics: 2010 pilot and 2017–2019 baseline. (XLSX)

S8 Table Cascade of hypertension care in 2010 and 2017–2019 for a 4-state pooled sample. (XLSX)

S9 Table Age-standardized mean systolic blood pressure (SBP) and diastolic blood pressure (DBP) among hypertensive adults aged 45+ years. (XLSX)

S10 Table State-level time trends in hypertension awareness, treatment, and control. (XLSX)

## Abbreviations

AME: average marginal effect
BP: blood pressure
DBP: diastolic blood pressure
LASI: Longitudinal Aging Study in India
OR: odds ratio
PM-JAY: Pradhan Mantri Jan Arogya Yojana
SBP: systolic blood pressure
