# Supplementary material for: Hypertension awareness, treatment, and control and their association with healthcare access in the middle-aged and older Indian population: A nationwide cohort study
Source: PLoS Med. 2022 Jan 4;19(1):e1003855. doi: 10.1371/journal.pmed.1003855 (PMC8726460; doi:10.1371/journal.pmed.1003855)
Supplement: S1 Appendix — (DOCX) [file pmed.1003855.s001.docx]

**Prospective Protocol**

Our goals are to: (1) develop and implement a nationally representative, longitudinal survey of aging, health, and retirement in India; (2) provide a foundation in India for innovative, rigorous, multidisciplinary studies of aging that will inform policy and advance scientific knowledge; and (3) provide data internationally harmonized with the Health and Retirement Study (HRS) and its sister studies around the world to enable cross-national comparative research on the influence of different institutions, cultures, and policies on aging.

**1. Sample design**

The target population for the LASI main wave will include all Indian adults and elderly men and women aged 45 and above and their spouses irrespective of age who reside in the households. There is no upper age limit for selection of respondents in LASI including biomarkers. The age of 45 is chosen to: (a) harmonize this survey with its sister HRS surveys in Asia^[[1]](#footnote-2)^; (b) allow measurement of pre-retirement behavior, as people often begin to change their labor market, health, and consumption behaviors before they retire; and c) determine early onset of chronic diseases among adults before reaching older ages. In accordance with the conventional practice for other population-based surveys, the sampling frame will include only the household population. Persons living in collective living arrangements, such as nursing homes, long-term dependent or care facilities, boarding houses, messes, hotels, residential hotels, rescue homes, jails, prisons, army camps, boarding schools, *ashrams*, etc. will not be considered as households, and will not be included in the survey.

The eventual observational unit of LASI is a LASI eligible household (LEH). The LASI eligible household (LEH) refers to the household having at least one member aged 45 and above. A ‘household’ is defined as comprising one or more individuals residing in a residential structure with cooking arrangements to prepare meals. Such individuals live together and take meals from a common kitchen unless the exigencies of work prevent any of them from doing so. There may be households of persons related by blood, of unrelated persons, or with a mix of both related and unrelated persons. In some cases, a group of people may live together in the same residential structure, but each person has a separate eating arrangement; such persons will be listed as separate one-person households. Nonresidential structures (shops, businesses, farm and cattle sheds, institutions, etc.) will be mapped but not listed in the LASI household sampling frame (universe).

A residential structure may have multiple households, each with a separate cooking arrangement. A residential structure which has multiple families is deemed as one household if food is prepared together in the same kitchen and shared by members. A household is deemed to have a head, a person who is recognized as head by the members of the household. The LEHs are those which have at least one member aged 45+. LASI will cover all men and women aged 45+, and their spouses even if they are outside this age range (<45) in the selected household. It implies that ***all married, non-married men and women aged 45+ and their spouses* in selected sample households will be interviewed.**

LASI (main waves) will be a biennial survey representative of the adult Indian population aged 45 and above. The research design to be adopted in the LASI main wave will be the longitudinal cluster sample research design (a panel study). LASI aims to follow a representative sample of the population aged 45 and above (including spouses irrespective of age) every two years for the next 25 years.

The main design considerations for LASI are:

- Need: Geographic limits.
  - Area sample units must be well-defined geographic/administrative units.
  - Sample units must contain sufficient household units to support a longitudinal survey.
  - The ultimate areas selected should not be sparsely spread with unreasonable travel requirements and should be accessible to teams for several follow-up surveys.

The following are the main characteristics of research design for the full waves of LASI:

- Representativeness – LASI aims to obtain all the indicators for the nation as a whole and for 29 states and two union territories. In addition, it aims to obtain indicators for each of the four metropolitan cities of Delhi, Kolkata, Mumbai and Chennai from the northern, eastern, western and southern parts of India, respectively.
- Panel Sample size – 50,000 households.
- Households with at least one person aged 45+ and their spouses irrespective of age.
- All persons aged 45+ and their spouses in sampled households.
- Longitudinal – 25 years.
- Sampling design- Multistage area probability sampling.

One of the major objectives of LASI is to estimate the prevalence of certain chronic diseases among the older population. The previous survey of the population aged 50 years and above has given estimates of different diseases among males and females: arthritis (18 percent), hypertension (17 percent), asthma (nine percent), diabetes (seven percent), angina (six percent), lung dysfunction (five percent), depression (four percent), and stroke (two percent) (Sage-India, 2010). With the assumption that: 1) **average prevalence of any disease is 5 percent**, and 2) design effect is 2, the required sample size to estimate prevalence with the margin error of 0.02 with a 95 percent confidence interval would be:

$$N=z_{1-\alpha/2}^{2}*\frac{p(1-p)}{d^{2}}*designeffect$$

Where: d=margin of error; p=proportion of population with particular characteristics; α=level of significance

$$={1.96}^{2}*\frac{(0.05*0.95)}{0.02*0.02}*2$$

= 912

= approximately 1000

Thus, a minimum sample of 1000 individuals aged 45 and above from each survey domain of each state is required to obtain reliable estimates of disease prevalence. Since, the states and union territories differ greatly in size, the sample will be allocated proportional to the size of the states and union territories. Additionally, LASI also aims to provide estimates for four metro cities of India, namely Delhi, Mumbai, Kolkata and Chennai, therefore, the sample size for states to which these metro cities belong will be increased accordingly. The major features of LASI sample size allocation are listed below:

- LASI national sample universe will include all households in **29 states and 2 union territories.**
- Nationally representative sample of **50,000 interviews** of persons aged 45+ years and their spouses in the **first wave.**
- The size will ensure sufficient sample for follow-up waves with significant attrition (mortality, mobility, non-response).
- 25+3 (10% addition for non-response) households will be selected interviews in each sampled area (site).
- 28 households in each site for obtaining 17 households (60% of 28 households) with at least one member age 45 and above.
- Out of 17 households, 9 are expected to have only one member age 45+ and the remaining 8 households are expected to have at least two members age 45+. The proportion having spouses less than age 45 is likely to be small.
- From a sample of 28 households in each ultimate area sampling unit, an average of 28 LASI eligible respondents are expected to be covered.
- With the assumption of 10% non-response, a sample of 28 households and consequently 28 respondents, an expected 25 interviews will be completed in each sample area.

The sampling procedure for the LASI survey is governed by the overall objectives of the survey. The main aim of LASI is to study the health status and social and economic well-being of the older population in India. In order to accomplish the objectives of LASI, the multistage area probability sampling procedure will be adopted to arrive at the eventual unit of observation (individuals aged 45 and above and their spouses irrespective of age). India is a union comprising 29 states and six union territories with a population of 1,210.2 million (Census of India, 2011). The states vary significantly with respect to geography, culture, population size, health conditions, demographic, and socio-economic characteristics. For administration and development planning, the states are further divided into districts, taluka/tehsil/blocks, and villages/cities/towns.

LASI sampling design consists of three stages of selection. At the first stage, sub-districts (tehsils/taluks) will be selected. At the second stage, villages/city wards will be selected from rural/urban areas of the selected tehsils. In rural areas, households will be selected from a sample of villages. However, sampling in urban areas involves one more stage. From each selected city ward one census enumeration block (CEB) will be selected, and a sample of households will be selected from a CEB. The first two stages (three stages in urban areas) pertain to selection of areas and the third stage involves selection of households from the sampled areas.

The sampling frame (universe) for the first stage will be the list of sub-districts as per 2011 census. The sampling frame for the second stage in the rural areas will be census villages from all the selected sub-districts in each stratum. In urban areas, the sampling frame will consist of all the city wards of all the selected sub-districts in each stratum. In urban areas, the list of CEBs in each ward will form sampling frame at the third stage. To obtain the sampling frame for the selection of households, a mapping and household listing operation will be carried out in the sampled second stage units, i.e. villages in rural areas and CEBs in urban areas. All the listed households in selected villages/CEBs will form the sampling frame for the selection of households.

Figure 1 (below) presents the hierarchy of the geographic administrative units that will be used for the primary, secondary, and ultimate third stage sample units. As illustrated, each state includes various tehsils/talukas (which can be compared to counties in the US).

Figure 1 LASI sampling design


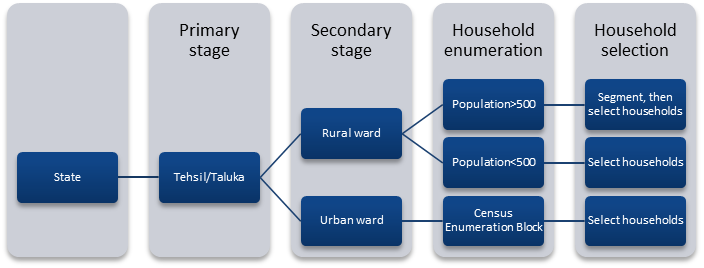


The first stage of sample selection involves selection of sub-districts (tehsils/taluks). The sampling procedure for selection of sub-districts is a stratified design. All the Sub-districts (tehsils/taluks) in the states will be stratified using a number of stratification criteria^[[2]](#footnote-3)^ in order to enhance the representativeness of the sample. From each stratum, a sample of sub-districts will be selected. The number of sub-districts to be selected from each stratum will be determined by the proportionate size of each stratum. From each stratum, primary sampling units (PSU) (taluks/tehsils) will be selected with equal probability sampling.

The selected sub-districts(tehsils/taluks) will form the universe for the second stage sample selection. This universe is further stratified into rural and urban. A proportionate sample of villages/urban wards will be sampled at the second stage using probability proportional to size (PPS) sampling procedure. The sampling units at the second stage will be called second stage units (SSUs).

Within the selected second stage sample units, LASI field staff will perform an enumeration of household units that will provide the frame of addresses or dwelling descriptions for the final stage sampling of households. Each urban ward is quite large and therefore, from each ward, one census enumeration block (CEB) comprising roughly 150 households will be selected at random for the enumeration step. The total number of households in the villages/cities/town wards varies from about 100 to more than 1,000. Therefore, in rural areas, if the number of households in a village is less than 500, the entire village will be covered for mapping and listing. If a village has more than 500 households, then segmentation will be done. That is, each large village will be divided into three or more segments of approximately equal size (e.g., 200-300 households). Of these segments, two will be selected randomly.

When the household listing is complete, a final stage sample of approximately 28 households will be randomly chosen from each selected second stage unit. Trained LASI interviewers will then visit each sample dwelling to complete the screening and proceed with the interview. Consistent with the probability sample design for LASI, no substitutions of sample addresses will be permitted. Dispositions of outcomes for all sample units will be carefully recorded to enable subsequent analysis of household contact, screening, eligibility, and interview rates.

Following data collection, analysis weights will be developed to account for the probability of selection both across and within states and variation in response rates across PSUs. Strata and cluster information that reflect the primary stratification (state and urban/rural areas within each state) and the primary stage clusters will also be added to each LASI dataset to enable design-based estimation and inference that reflects the full complexity (stratification, clustering, and weighting) of the LASI area probability sample of households.

**2. Survey instrument**

The pilot study offered invaluable lessons for planning the baseline instrument. The LASI instrument comprises the household survey (carried out once for each household by interviewing the key informant) and the individual survey (which the interviewer carries out for each respondent). We aim to keep the same interview length for the baseline as the pilot study: approximately 30 minutes for the household interview, one hour for the individual interview, and 20 minutes for the biomarker protocol.

The household survey starts with the cover screen, containing questions about the demographic composition of the household and identifying key informants for the following household modules: (1) housing and environment section, consisting of questions about the household’s physical dwelling, residential history, and physical and social characteristics of neighborhood; (2) income, which attempts to capture the complete income of all household members, as well as remittances from non-household members; (3) assets and debts, including detailed questions appropriate to the Indian context; and (4) consumption, designed to collect data on both market-purchased and home-produced consumption at the household level.

The individual survey covers: (1) demographics, including birth date, sex, religion, caste, language, marital status, literacy, education, and questions designed to approximate age when necessary; (2) family and social networks, asking detailed questions about all immediate family members, including parents, children, and siblings, both alive and deceased, social activities, and psychosocial measures (including life satisfaction, emotional proximity, and social status) and social connectedness, an innovative module introduced by LASI; (3) health, including questions about overall health and specific diseases, health events, cognitive and physical function tests, mental health, health behaviors, and anchoring vignettes; (4) health insurance and health care utilization, designed to capture access to and use of different types of health care providers; (5) work and employment, including questions about current job (including self-employment and (subsistence) agriculture) and employment history; (6) pensions, designed to reflect India’s growing pension scheme; and (7) expectations, i.e., questions developed to measure subjective probabilities of survival to specific ages, of working to specific ages, and of health conditions for illiterate survey respondents, using visual aids.

While more than 90% of the instrument will remain the same, as our pilot study has shown our instrument to work effectively, we will fine-tune the instrument based on further analyses of pilot data. First, we will further harmonize the instrument with other HRS-type studies. *For the consumption and asset modules, we will improve cross-country comparability with developing countries like China and Indonesia. For the cognition module, we are collaborating with other survey teams (i.e., IFLS, Mexican Health and Aging Study (MHAS)) in designing harmonized cognitive tests that are suitable for illiterate people. We are also working with HRS and the English Longitudinal Ageing Study (ELSA) team to measure time use and well-being*. Our pilot study collected information about limitations in activities of daily living (ADL), but did not collect any information about mobility. Following other HRS surveys, we will start with mobility questions and ask about ADL limitations for those with mobility limitations.

Second, we will streamline the questionnaire. By examining the distribution of responses, we will identify questions or response categories that can be combined; for example, when we ask about specific types of social activities and consumption items, we can, by identifying and merging the items with rare responses, reduce interview time and therefore respondent burden. Similarly, we will shorten psychosocial measures based on item-response theory (IRT). We have included a number of psychosocial measures (e.g., the 20-item Center for Epidemiologic Studies Depression Scale, or CESD); we will analyze the properties of these psychosocial measures and shorten the list of questions, using IRT to reduce respondent burden.

Third, we will clarify the wording of questions. During the pilot study, interviewers kept records of difficult-to-communicate questions, and we will address all the issues raised. Using time-stamped data, we will examine questions that had unusually long administration times and clarify their wording. During the pilot, we learned that visual aids (e.g., index cards illustrating the responses, such as source of drinking water or toilet type) are particularly effective for illiterate populations, so we will prepare more such cards to facilitate communication.

Fourth, we will update all threshold values for unfolding bracket questions, taking inflation into account. Furthermore, the pilot data have given us the distribution of income, assets, debts, and consumption, and using this information, we will create more efficient threshold values.

Fifth, we will introduce additional questions about frailty, cognition, and discrimination, with guidance from experts in those areas. The pilot study found that about 47% of the sample never attended formal schooling and, therefore, may never have learned to count, which highlights the importance of appropriate cognitive tests for unschooled populations. Dr. Mary Ganguli helped us identify cognitive tests designed for such populations. We will, however, ensure that we are measuring both ends of cognitive extremes: incorporating recent innovations in HRS cognitive tests, we will consider adopting adaptive testing to measure higher-order cognitive ability in consultation with Dr. Jack McArdle. Similarly, we will introduce *additional psychosocial measures, such as perceived discrimination*, in consultation with Dr. Jacqui Smith. We will also explore other areas, especially strengthening physical and environmental measures and lineage or family medical history.

As with the pilot instrument, we will first finalize and pretest the English version of the instrument and then translate it into local languages. We translated the pilot instrument into Hindi, Kannada, and Malayalam. We plan to translate the baseline instrument into as many local languages as necessary. We expect that this will require 10 translated versions of the survey. To ensure the validity of the translations for the full-scale LASI survey instrument, we plan to back-translate the entire instrument.

LASI is designed as a biennial survey, tracking and re-interviewing panel households and respondents. We aim to develop two types of longitudinal interviews: (a) a follow-up interview with panel households and individual respondents within those households; and (b) an exit interview with family or friends of any deceased respondents. In developing a follow-up interview, we will first consult with other HRS-type surveys and learn about the extent of pre-loaded information and how to reconcile inconsistencies between past and current survey responses. In designing the Wave 2 instrument, we do not want to burden respondents by repeating questions from the baseline survey. However, the follow-up interview presents an opportunity to correct any inaccurate information from a prior wave. Carefully preloaded information can reduce interview burden and increase the quality of the interview. We will develop a Wave 2 instrument and test it on the pilot sample. For the exit interview, we will first explore the possibility of linking to administrative mortality records. As with the follow-up interview, we will harmonize with exit interviews from other HRS-type surveys, while taking the Indian context into full consideration.

**3. Biomarker protocol**

Drawing from the pilot protocol, we aim to collect anthropometry (i.e., height, weight, and hip and waist circumferences), functional assessment (i.e., blood pressure, lung function, vision test, gait speed, grip strength, and balance tests), and molecular biomarkers through the collection of dried blood spots (DBS). *We will also use CardioChek and A1cNow to measure lipids and HbA1c, given their proven effectiveness in field settings.* Each respondent participating in the biomarker module will receive a report on the measurements taken (but not on the DBS, unless abnormalities are revealed by the subsequent assays). If results are abnormal, respondents will be advised to seek medical care and will be given a referral letter to a local health center or doctor.

Our preliminary analyses of pilot data demonstrated our ability to collect high-quality biometric data from a large number of respondents. These analyses also show that such data appear to correlate meaningfully with other indicators of health. We must note, however, that a tendency for some interviewers to omit digits in the recording of pulse indicates that there is room to improve interviewer training and related materials.

To enhance the quality of biomarker data collected, we propose to: (1) improve the interviewer manual; (2) improve interviewer training led by the biomarker co-investigator, Dr. Peifeng Hu; and (3) standardize the training protocol for state-level supervisors and individual interviewers.

Following the standard DBS collection protocol implemented for the pilot, we will collect five dried blood spots by administering a finger prick to each respondent. The DBS samples are allowed to air-dry on a rack for at least four hours or overnight; the dry samples are then placed in separate plastic ziplock bags with desiccants, which keep the samples dry, and put into a larger plastic ziplock bag and a paper envelope to be delivered by BlueDart, a certified blood courier, to the storage facility NARI.

The field teams prepare four copies of DBS transmittal sheets for each Primary Sampling Unit (PSU). Three copies will accompany the DBS sample box (a properly packed thermo box that can store about 30 DBS samples), and one will be kept by the field agency. NARI keeps one copy of the transportation sheet, a second is sent to where the DBS sample was collected and shipped, and a third is sent to Dr. P. Arokiasamy at IIPS for confidential record-keeping. At NARI, the number and quality of DBS samples are verified and documented.

The DBS samples are stored in designated, serially numbered racks in the freezer cabinets in locked and restricted areas at NARI. NARI has four backup generators linked to all equipment, including the freezer cabinets. NARI maintenance staff continually monitor the freezing facility. NARI has an alarm system to notify researchers of any electrical failures and a well-developed system to protect the safety and confidentiality of the samples. Assuming proper collection and storage, these DBS can be stored for decades and used for additional bioassays in the future, including ones not currently existing or anticipated.

As an extension of the pilot study, we have been awarded an R21 (1R21AG034443-01A2) to test the DBS for the following molecular biomarkers and to examine their relationship to other self-reported health outcomes and socioeconomic status: C-reactive protein (CRP, a marker of inflammation), glycosylated hemoglobin (HbA1c, a marker of glucose metabolism), hemoglobin (Hb, a marker of anemia), and Epstein-Barr virus (EBV) antibodies (a marker of cell-mediated immune function). After successful completion of this R21 for biomarkers testing, we will seek similar funding for assays to be conducted on DBS collected in Wave 1 and Wave 2 of the full-scale LASI. As part of a separate future funding application, we plan to develop new protocols for markers that would hold special importance in India, such as measures of malnutrition.

**4. Fieldwork and management**

Before initiating fieldwork, we will meet with local officials and community leaders, distribute printed informational brochures, and publish press releases in local newspapers to heighten awareness and increase response rates, as we did in the pilot. To maximize cooperation of sampled households and individuals, we will prepare materials explaining our purpose, mechanisms for protecting their privacy, the safety of health assessments, etc., and will distribute them before beginning the survey, as detailed in the Human Subjects Protection component of the application.

IIPS will subcontract the fieldwork to state-level implementing agencies; Dr. Arokiasamy will oversee the process of fieldwork agency selection and interviewer recruitment and training. To ensure uniformity in data collection, IIPS has developed well-established mapping and listing protocols and training manuals, and will host workshops to train trainers prior to data collection. The manuals will consist of instructions to the interviewers regarding interviewing techniques, field procedures, and the method of asking questions consistently and using CAPI to record answers. Interviewer training will also cover mapping and listing of survey areas, objectives and features of the survey instrument, collection of biomarkers, the proper management of biohazardous waste, and monitoring and supervision procedures. Those trained at each workshop will transfer skills to their field staff in each state according to standard IIPS procedures. Interviewers will obtain consent from prospective respondents in a regimented manner in accordance with international standards (see Appendix B for Harvard, IIPS, and USC IRB approvals; and approved consent materials).

As with the pilot study, we will bring the HRS field team supervisors to India to monitor and ensure the quality of interview training. IIPS will coordinate the field teams, with each team consisting of four interviewers (two male, two female) and two supervisors. Each interviewer will be trained in administering the questionnaire and recording biomarkers. One supervisor will closely monitor interviews, biomarker collection, and adherence to CAPI protocol; the other supervisor will serve as the team leader and will be responsible for coordinating logistics, allocating the daily workload for each interviewer, and communicating with the state-level supervisor.

During the pilot study, we developed a protocol for quality control that will be closely followed. Evaluation of data quality will be monitored by a research officer and a health coordinator from IIPS, who will conduct spot checks and back-checks. Spot checks refer to monitoring fieldwork during the face-to-face interview. The team supervisor will make random site visits for roughly 10% of individual interviews to check the quality of the fieldwork. IIPS field supervisors will also conduct random spot checks during which they will observe and help improve interview techniques and the recording of responses. Back-checks refer to follow-up calls to respondents to check the validity of responses after the interview. Field supervisors, field coordinators, and IIPS research officers will conduct back-checks on 5% of cases, randomly selected. The IIPS PI and coordinators will also make periodic visits to the field to undertake spot observations and random back checks.

To strengthen data quality, we will keep records of the date and time of each contact at a given address and the results of the contact through a sample management system. IIPS will use this record to help verify the responses and evaluate interviewer performance. We will also identify interviewer characteristics (e.g., age, gender, and interview experience), which will be used to analyze their potential impact on responses.

To guard against data loss, we will install backup servers at both HSPH and USC to which field data will be uploaded daily or as often as local internet access permits, but no less than once per week. In addition, laptops will be outfitted with encrypted flash drives with software to facilitate frequent backups. Each institution will assign staff to monitor the data uploads and check fieldwork progress in real time.

During the pilot study, the USC research team developed an automated quality control protocol to examine fieldwork progress and identify unusual response patterns (e.g., inconsistent answers to reverse coded items, and extreme values), incomplete recording (e.g., not including at least two digits for pulse), and interview time (e.g., unusually short or lengthy interview), signaling potential problems in fieldwork implementation.

In preparing for Wave 2, we propose to collect mobile phone numbers from respondents during Wave 1, along with contact information of friends, employers, and non-coresiding family members. Through this pretest of the longitudinal study, we will identify remaining challenges and devise appropriate methods to minimize attrition. *As Thomas et al (2012)^17^ found, the respondent characteristics associated with attrition might differ between those who were not able to be contacted in the subsequent wave and those who refused to participate in the interview. Therefore, we will carefully examine the characteristics of those who refused, relocated, and participated in the interview. To minimize contact failure, we will collect contact information as described above. To minimize the refusal rate, we will design and examine the effectiveness of incentive schemes for the follow-up interview.*

*Following the IFLS’s effective strategies for tracking (less than 6% attrition between five-year waves), we will separate out long-distance tracking from local tracking. ‘Local’ will be defined as households that moved within 45 minutes by car, bus, or train. The original field team will be responsible for tracking and interviewing locally relocated households and respondents. IIPS will coordinate long distance tracking and relay tracking information to the field team that is closest to that person/household. To encourage successful tracking, financial rewards will be offered to teams that achieve the highest rates of tracking success.*

**5. Survey technology and database management**

As in the pilot, we will use CAPI for the baseline and follow-up interviews. This method requires that field teams be outfitted with laptop computers pre-loaded with survey questions asked to respondents in a face-to-face interview. Field teams input responses directly into the computer. The use of CAPI allows for cross-checking of data in real-time, thereby minimizing data entry errors and ensuring internal consistency.

The USC team has spearheaded the development of a comprehensive information system, MMIC™ (Multimode Interviewing Capability). MMIC™ was used to program the CAPI survey for the pilot. It was also used to manage the entire data collection process from questionnaire design, sample management, and fieldwork monitoring to final dataset production. The USC team will process the collected CAPI data every week during the fieldwork period and immediately after its completion and provide Stata datasets to IIPS and HSPH for cleaning and coding.

To ensure efficient and rigorous data cleaning, coding, and codebook preparation, both HSPH and IIPS will undertake data cleaning and coding independently, and the resulting data sets will be compared. Any revealed errors will be resolved, with further (and retroactive) data cleaning and coding carried out as necessary. Errors that cannot be resolved will be checked through revisits to households. In this way we will ensure the integrity and information content of the dataset. The cleaned dataset will be sent to USC for reformatting, exploratory data analysis, and imputation, as discussed below.

**6. Exploratory data analysis and imputations**

In preparation for public data release, we first plan to conduct the following exploratory data analyses. First, the USC research team will examine reasons for non-response (e.g., contact failure, refusal). We will compile cause-specific non-response data (including details regarding sex, age, and housing conditions of non-respondents), analyze item non-responses, evaluate interviewer variance with respect to patterns of response and non-response, analyze response effects by item and by subgroup, assess the severity of response effects and propose remedies, and evaluate willingness for proxy interviews (i.e., by other household members).^[[3]](#endnote-2)^ These analyses will be documented in reports that will be used both to improve procedures for the next wave and to monitor the quality of the completed fieldwork.

Missing values will be imputed by the USC research team using regression-based imputation methods that have been applied in similar imputations for the HRS. Dr. Erik Meijer, a co-investigator at the USC LASI team, will lead this effort, utilizing his experience as director of the USC HRS imputation team. The same USC programming team that has created imputation programs for the HRS will carry out imputation for the LASI data, in close partnership with IIPS.

We will carefully check the individual and household data for internal consistency. The Harvard and IIPS research teams will also compare the descriptive statistics and patterns with those from other sources of information about population, health, and retirement, both in India and other developing countries.

We will prepare a descriptive statistical analysis of the results. For each variable that measures some aspect of social, economic, or physical well-being (including an array of biomarkers), we will examine the variation across states, age, sex, living arrangements, urban/rural residence, and socioeconomic status. In addition, psychometric properties of multi-item psychological measures, such as perceived employment characteristics (control/demand and effort/reward),^[[4]](#endnote-3)^ emotional proximity, and life satisfaction, will be also investigated, measuring their internal consistency.

**7. Substantive data analysis and potential for comparative research**

We plan to conduct substantive data analyses on: (1) the health and well-being of older adults, including key components of the disease burden in India (i.e., hypertension and diabetes), with attention to subpopulation differences and self-report biases; (2) health care access and utilization and undiagnosed diseases, using both self-reports and biomarkers; (3) cognitive and physical functioning, based on self-reports and performance tests; (4) labor-force participation in both formal and informal sectors of the economy; (5) old-age income dependency, sources of household income, pension holdings, and remittances; (6) asset portfolios, financial asset holdings, and inflationary expectations; and (7) social environments and behaviors, such as networks, support, conflict, and activities. **It is important to emphasize that the primary goal of LASI is to produce and disseminate high-quality data for public use, and that the LASI data offer many scientific opportunities that go beyond our data analysis plan.**

LASI’s harmonized research design and instrument present enormous potential for comparative research. Within the realm of comparative analyses, we propose to do the following: (1) Examine obesity and cardiovascular health and their risk factors, especially comparing two developing countries in epidemiological transition, China and India, using CHARLS and LASI, respectively. (2) Investigate how economic development and access to health care impacts the prevalence of undiagnosed diseases; using self-reported and directly assessed markers of hypertension and diabetes, we will examine the extent of undiagnosed diseases. By making cross-country comparisons across the U.S., Germany, Mexico, China, and India, using HRS, SHARE-Germany (only German SHARE collects biomarkers at this time), the Mexican Health and Aging Study (MHAS), CHARLS, and LASI, we will further investigate how individual socioeconomic status, the level of economic development, and the provision of health insurance influence non-diagnosis and non-treatment of diseases. And (3) examine the effect of education on cognitive functioning in old age, using HRS, ELSA, SHARE, CHARLS, and LASI, exploiting the variations in compulsory education mandates across countries and within countries over time.

**8. Data dissemination**

LASI is committed to providing publicly available, high-quality data to the international scientific community, stimulating international comparative studies. *We released the pilot data to the public in January 2012, within one year of completing pilot data collection. As of June 2012, more than 100 unique users have downloaded the pilot data. In addition, we have supplied the data directly to numerous others. The full-scale LASI data will be also de-identified and released to the public via the Gateway to Global Aging Data within one year after completing the fieldwork, together with the survey instrument, interviewer manuals, descriptive data analyses, reports by the three partner organizations, and manuscripts and papers based on LASI data.* Researchers and policy analysts may access the data at no charge, and in a range of formats (e.g., Stata, SAS, SPSS). The descriptive information needed to understand the data will be in English. The data will be provided in a user-friendly format and will include some auxiliary variables. The website will contain full details of the data content, access arrangements, survey instruments, and other documentation. To widely disseminate LASI data, we plan to contact individual researchers, institutions, and organizations that might have a particular interest in such data.

After each wave, we will prepare a comprehensive report, summarizing the descriptive findings about health and retirement behavior with contextual information on India, similar to the key findings book prepared by other HRS-type surveys (e.g., ELSA, SHARE). This report will be shared with state and national policy makers, government program managers, researchers, and other key stakeholders to increase the awareness of what LASI data can offer, thereby encouraging data usage. We will also aim to conduct special sessions at meetings of professional conferences, such as the Indian Association for the Study of Population and the Population Association of America, to discuss LASI with prospective users. After public release, we will look for additional funds to organize a conference at HSPH, IIPS, or USC for presenting papers using these data; *we are already planning to submit papers to a Harvard-Stanford conference on population aging in China and India, to be held in March 2013. This conference will present an opportunity to compare LASI and CHARLS data, and will lead to a special issue of a new Elsevier journal: Journal of the Economics of Ageing.* We will also distribute fliers at other major international conferences to inform prospective users of these data.

1. KLoSA and CHARLS also employ the age cutoff of 45, whereas HRS, ELSA, and SHARE employ the age cutoff of 50. Because of the shorter life expectancy in India, we chose the age cutoff of 45. [↑](#footnote-ref-2)
2. All the sub-districts in each state/union territory will be stratified by the following criteria: 1) sub-district size, 2) percentage of urban population, 3) female literacy, and 4) percentage of Scheduled caste/Scheduled tribe population. Then, a proportionate sample of sub-districts (taluks/tehsil) will be selected from each stratum independently. [↑](#footnote-ref-3)
3. Groves, R. M., D. A. Dillman, et al., Eds. (2001). *Survey Nonresponse*. New York, John Wiley & Sons. [↑](#endnote-ref-2)
4. Schwartz, J. E., C. Pieper, et al. (1988). "A procedure for linking psychosocial job characteristics data to health surveys." *American Journal of Public Health* (78): 904-909. [↑](#endnote-ref-3)
