# Supplementary material for: Hypertension awareness, treatment, and control and their association with healthcare access in the middle-aged and older Indian population: A nationwide cohort study
Source: PLoS Med. 2022 Jan 4;19(1):e1003855. doi: 10.1371/journal.pmed.1003855 (PMC8726460; doi:10.1371/journal.pmed.1003855)
Supplement: S1 Code — (DOCX) [file pmed.1003855.s002.docx]

************************************************************************************************************

*Summary: Produces analytic dataset for 2010 Pilot for PLOS Medicine Hypertension manuscript

************************************************************************************************************

*****load variables from pilot datasets*****

***Harmonized LASI pilot***

use hhidpnc inw0 r0state r0pwtresp r0swtresp r0agey raeduc_l ragender r0lvreg r0hibpe h0ctot h0hhres using "H_LASI_pilot.dta"

gen prim_key = hhidpnc

destring hhidpnc, gen(hhidpn)

***merge with pooled 4-state weight***

merge 1:1 hhidpn using "r0pwtresp4.dta", keepusing(r0pwtresp4) nogen

***merge raw LASI pilot***

merge 1:1 prim_key using "LASI-Pilot_all.dta", keepusing(ht005 hc106 hc109s4)

***merge raw LASI biomarkers***

merge 1:1 prim_key using "LASI-Pilot_biomarker_all.dta", keepusing(bm008 bm009 bm013 bm014 bm018 bm019) nogen

***defining value labels***

label define yesnopilot 0 "0.no" 1 "1.yes"

label define agecat 1 "1.45-54" 2 "2.55-64" 3 "3.65-74" 4 "75+"

label define agecat5 1 "1.45-49" 2 "2.50-54" 3 "3.55-59" 4 "4.60-64" 5 "5.65-69" 6 "6.70-74" 7 "7.75+"

label define educcat 1 "1.no education" 2 "2.primary school" 3 "3.any secondary school"

label define stage 1 "1.normal" 2 "2.prehibp" 3 "3.stage 1 hibp" 4 "4.stage 2 hibp" 5 "5.stage 3 hibp"

label define tertile 1 "1.1st tertile" 2 "2.2nd tertile" 3 "3.3rd tertile"

***recode urban/rural***

gen r0rural = .

replace r0rural = 0 if r0lvreg==1

replace r0rural = 1 if r0lvreg==2

label variable r0rural "r0rural:w0 lives in rural"

label values r0rural yesnopilot

***10 year age groups***

gen r0agegrp = .

replace r0agegrp = 1 if inrange(r0agey,45,54)

replace r0agegrp = 2 if inrange(r0agey,55,64)

replace r0agegrp = 3 if inrange(r0agey,65,74)

replace r0agegrp = 4 if inrange(r0agey,75,200)

label variable r0agegrp "r0agegrp:w0 10 year age groups"

label values r0agegrp agecat

***5 year age groups***

gen r0agegrp5 = .

replace r0agegrp5 = 1 if inrange(r0agey,45,49)

replace r0agegrp5 = 2 if inrange(r0agey,50,54)

replace r0agegrp5 = 3 if inrange(r0agey,55,59)

replace r0agegrp5 = 4 if inrange(r0agey,60,64)

replace r0agegrp5 = 5 if inrange(r0agey,65,69)

replace r0agegrp5 = 6 if inrange(r0agey,70,74)

replace r0agegrp5 = 7 if inrange(r0agey,75,200)

label variable r0agegrp5 "r0agegrp5:w0 5 year age groups"

label values r0agegrp5 agecat5

***education levels***

gen r0educgrp = .

replace r0educgrp = 1 if raeduc_l==0

replace r0educgrp = 2 if inrange(raeduc_l,1,2)

replace r0educgrp = 3 if inrange(raeduc_l,3,9)

label variable r0educgrp "r0educgrp:w0 education level"

label values r0educgrp educcat

***per capita consumption tertiles***

gen hh0cperc = h0ctot/h0hhres

xtile h0contert = hh0cperc, nquantiles(3)

label variable h0contert "h0contert:w0 pcc tertiles"

label values h0contert tertile

***personally covered by any health insurance***

gen r0personalhi = .

replace r0personalhi = 0 if hc106==2

replace r0personalhi = 1 if hc106==1

label variable r0personalhi "r0personalhi:w0 has health ins"

label values r0personalhi yesnopilot

***replace observations where systolic<diastolic***

*first measurement

replace bm008 = . if bm009>bm008 & !mi(bm008) & !mi(bm009)

replace bm009 = . if bm009>bm008 & !mi(bm008) & !mi(bm009)

*second measurement

replace bm013 = . if bm014>bm013 & !mi(bm013) & !mi(bm014)

replace bm014 = . if bm014>bm013 & !mi(bm013) & !mi(bm014)

*third measurement

replace bm018 = . if bm019>bm018 & !mi(bm018) & !mi(bm019)

replace bm019 = . if bm019>bm018 & !mi(bm018) & !mi(bm019)

***Average blood pressure-systolic (2nd & 3rd readings)

gen r0systo = .

replace r0systo = (bm013 + bm018)/2 if inrange(bm013,16,300) & inrange(bm018,16,300)

*replacing with average of 1st and 2nd readings if missing 3rd (4 obs)

replace r0systo = (bm008 + bm013)/2 if inrange(bm008,16,300) & inrange(bm013,16,300) & mi(bm018) & mi(r0systo)

*replacing with 1st reading if missing 2nd and 3rd (3 obs)

replace r0systo = bm008 if inrange(bm008,16,300) & mi(bm013) & mi(bm018) & mi(r0systo)

label variable r0systo "r0systo:w0 r average blood pressure measure (systolic) 2 & 3"

***Average blood pressure-diastolic (2nd & 3rd readings)

gen r0diasto = .

replace r0diasto = (bm014 + bm019)/2 if inrange(bm014,1,150) & inrange(bm019,1,150)

***replacing with average of 1st and 2nd readings if missing 3rd (not needed)

*replace r0diasto = (bm009 + bm014)/2 if inrange(bm009,1,150) & inrange(bm014,1,150) & mi(bm019) & mi(r0diasto)

***replacing with 1st reading if missing 2nd and 3rd (not needed)

*replace r0diasto = bm009 if inrange(bm009,1,150) & mi(bm014) & mi(bm019) & mi(r0diasto)

label variable r0diasto "r0diasto:w0 r average blood pressure measure (diastolic) 2 & 3 "

***measured hibp***

gen r0mhibp = .

replace r0mhibp = 0 if inrange(r0systo,0,139.9) & inrange(r0diasto,0,89.9)

replace r0mhibp = 1 if (inrange(r0systo,140,300) | inrange(r0diasto,90,150)) & !mi(r0systo) & !mi(r0diasto)

label variable r0mhibp "r0mhibp:w0 has measured hypertension"

label values r0mhibp yesnopilot

***stage measured hibp***

gen r0smhibp = .

replace r0smhibp = 1 if inrange(r0systo,0,129.99) & inrange(r0diasto,0,84.99)

replace r0smhibp = 2 if (inrange(r0systo,130,139.99) | inrange(r0diasto,85,89.99)) & !mi(r0systo) & !mi(r0diasto)

replace r0smhibp = 3 if (inrange(r0systo,140,159.99) | inrange(r0diasto,90,99.99)) & !mi(r0systo) & !mi(r0diasto)

replace r0smhibp = 4 if (inrange(r0systo,160,179.99) | inrange(r0diasto,100,109.99)) & !mi(r0systo) & !mi(r0diasto)

replace r0smhibp = 5 if (inrange(r0systo,180,300) | inrange(r0diasto,110,150)) & !mi(r0systo) & !mi(r0diasto)

label variable r0smhibp "r0smhibp:w0 stage measured hypertension"

label values r0smhibp stage

***total hibp***

gen r0tothibp = .

replace r0tothibp = 0 if r0hibpe==0 & r0mhibp==0

replace r0tothibp = 1 if (r0hibpe==1 | r0mhibp==1) & !mi(r0hibpe) & !mi(r0mhibp)

label variable r0tothibp "r0tothibp:w0 has total hypertension"

label values r0tothibp yesnopilot

***diagnosed among hypertensives***

gen r0diaghibp = .

replace r0diaghibp = 0 if r0tothibp==1

replace r0diaghibp = 1 if r0hibpe==1

label variable r0diaghibp "r0diaghibp:w0 diagnosed if hypertensive"

label values r0diaghibp yesnopilot

***taking medication among hypertensives***

gen r0hibprx = .

replace r0hibprx = 0 if r0tothibp==1

replace r0hibprx = 1 if ht005==1

label variable r0hibprx "r0hibprx:w0 on medication if hypertensive"

label values r0hibprx yesnopilot

***bp controlled among hypertensives***

gen r0hibpcon = .

replace r0hibpcon = 0 if r0tothibp==1

replace r0hibpcon = 1 if r0mhibp==0 & r0tothibp==1

label variable r0hibpcon "r0hibpcon:w0 bp controlled if hypertensive"

label values r0hibpcon yesnopilot

***hypertensive sample***

gen samplehyp0 = 0

replace samplehyp0 = 1 if !mi(r0agegrp5) & !mi(ragender) & !mi(r0educgrp) & !mi(h0contert) & ///

!mi(r0rural) & !mi(r0personalhi) & r0tothibp==1

***whole non-missing sample***

gen samplewhl0 = 0

replace samplewhl0 = 1 if !mi(r0agegrp5) & !mi(ragender) & !mi(r0educgrp) & !mi(h0contert) & ///

!mi(r0rural) & !mi(r0personalhi) & !mi(r0tothibp)

***save dataset***

save "pilot", replace

clear

************************************************************************************************************

*Summary: Produces analytic dataset for 2017-19 Baseline for PLOS Medicine Hypertension manuscript

************************************************************************************************************

***load variables from baseline datasets***

***Harmonized LASI w1***

use prim_key hhid inw1 ragender r1agey raeduc_l hh1state hh1rural r1wtresp hh1cperc ///

r1hibpe r1systo r1diasto r1systo1 r1systo2 r1systo3 r1diasto1 r1diasto2 r1diasto3 using "H_LASI.dta"

***merge with 4-state pooled weight data***

merge 1:1 prim_key using "r1pwtresp4.dta", keepusing(r1pwtresp4) nogen

***merge with raw LASI individual data***

merge 1:1 prim_key using "lasi_ind2.dta", keepusing(ht002c ht002d ht002b_year ht002b_age hb001 hb003 hb003_a hb006 hc102 ///

hc106_year hc002s1 hc002s2 hc002s3 hc002s4 hc002s5 hc002s6 hc002s7 hc002s8 hc002s9 hc002s10 ///

hc002s11 hc002s12 hc002s13 hc002s14 hc002s15 hc002s16 hc003s1 hc003s2 hb215 ssuid) gen(merge_ind)

***merge with raw LASI community data***

merge m:1 ssuid using "Final_clean_Community_data_24_2_21.dta", keepusing(state rc001a_a rc001a_b rc001a_c rc001a_d ///

rc001a_e rc001a_f rc001a_g rc001a_h rc001a_i rc001a_j rc001a_k ua011a_a ua011a_b ua011a_c ua011a_d ///

ua011a_e ua011a_f ua011a_g ua011a_h ua011a_i ua011a_j ua011a_k ua011a_l ua011a_m ///

uc020a_a uc020a_b uc020a_c uc020a_d uc020a_e uc020a_f uc020a_g uc020a_h uc020a_i uc020a_j uc020a_k uc020a_l uc020a_m uc020a_n) gen(merge_comm)

keep if merge_comm==3 //we lose 1

drop merge_ind merge_comm

***defining value labels***

label define agecat 1 "1.45-54" 2 "2.55-64" 3 "3.65-74" 4 "75+"

label define agecat5 1 "1.45-49" 2 "2.50-54" 3 "3.55-59" 4 "4.60-64" 5 "5.65-69" 6 "6.70-74" 7 "7.75+"

label define educcat 1 "1.no education" 2 "2.primary school" 3 "3.any secondary school"

label define yesnobase 0 "0.no" 1 "1.yes"

label define stage 1 "1.normal" 2 "2.prehibp" 3 "3.stage 1 hibp" 4 "4.stage 2 hibp" 5 "5.stage 3 hibp"

label define tertile 1 "1.lowest" 2 "2.middle" 3 "3.highest"

***10 year age groups***

gen r1agegrp = .

replace r1agegrp = 1 if inrange(r1agey,45,54)

replace r1agegrp = 2 if inrange(r1agey,55,64)

replace r1agegrp = 3 if inrange(r1agey,65,74)

replace r1agegrp = 4 if inrange(r1agey,75,200)

label variable r1agegrp "r1agegrp:w1 10 year age groups"

label values r1agegrp agecat

***5 year age groups***

gen r1agegrp5 = .

replace r1agegrp5 = 1 if inrange(r1agey,45,49)

replace r1agegrp5 = 2 if inrange(r1agey,50,54)

replace r1agegrp5 = 3 if inrange(r1agey,55,59)

replace r1agegrp5 = 4 if inrange(r1agey,60,64)

replace r1agegrp5 = 5 if inrange(r1agey,65,69)

replace r1agegrp5 = 6 if inrange(r1agey,70,74)

replace r1agegrp5 = 7 if inrange(r1agey,75,200)

label variable r1agegrp5 "r1agegrp5:w1 5 year age groups"

label values r1agegrp5 agecat5

***education levels***

gen r1educgrp = .

replace r1educgrp = 1 if raeduc_l==0

replace r1educgrp = 2 if inrange(raeduc_l,1,2)

replace r1educgrp = 3 if inrange(raeduc_l,3,9)

label variable r1educgrp "r1educgrp:w1 education level"

label values r1educgrp educcat2

***per capita consumption***

xtile h1contert = hh1cperc, nquantiles(3)

label variable h1contert "h1contert:w1 pcc tertiles"

label values h1contert tertile

***personally covered by any health insurance***

gen r1personalhi = .

replace r1personalhi = 0 if hc102==2

replace r1personalhi = 1 if hc102==1

label variable r1personalhi "r1personalhi:w1 has health ins"

label values r1personalhi yesnobase

***public health center in community***

*rural - b.phc

*urban (ward) - c.urban community health center

*urban (ceb) - c.phc

*rural & ceb (if no ceb then ward)

gen r1phcchf = .

replace r1phcchf = 0 if hh1rural==1 & (rc001a_b==2) //rural

replace r1phcchf = 0 if hh1rural==0 & (uc020a_c==2) //urban ceb

replace r1phcchf = 1 if hh1rural==1 & (rc001a_b==1) //rural

replace r1phcchf = 1 if hh1rural==0 & (uc020a_c==1) //urban ceb

replace r1phcchf = 1 if hh1rural==0 & mi(r1phcchf) & (ua011a_c==1) //urban ward

replace r1phcchf = 0 if hh1rural==0 & mi(r1phcchf) & (ua011a_c==0) //urban ward

label variable r1phcchf "r1phcchf:w1 has access to public health center"

label values r1phcchf yesnobase

***private health facility in community***

*rural - d.private clinic, e.private nursing home or hospital

*urban (ward) - e.private clinic, f.private hospital

*urban (ceb) - e.private clinic, f.private hospital

*rural & ceb (if no ceb then ward)

gen r1prichf = .

replace r1prichf = 0 if hh1rural==1 & (rc001a_d==2 | rc001a_e==2) //rural

replace r1prichf = 0 if hh1rural==0 & (uc020a_e==2 | uc020a_f==2) //urban ceb

replace r1prichf = 1 if hh1rural==1 & (rc001a_d==1 | rc001a_e==1) //rural

replace r1prichf = 1 if hh1rural==0 & (uc020a_e==1 | uc020a_f==1) //urban ceb

replace r1prichf = 1 if hh1rural==0 & mi(r1prichf) & (ua011a_e==1 | ua011a_f==1) //urban ward

replace r1prichf = 0 if hh1rural==0 & mi(r1prichf) & (ua011a_e==0 | ua011a_f==0) //urban ward

label variable r1prichf "r1prichf:w1 has access to private health facility"

label values r1prichf yesnobase

***measured hibp***

gen r1mhibp = .

replace r1mhibp = 0 if inrange(r1systo,0,139.99) & inrange(r1diasto,0,89.99)

replace r1mhibp = 1 if (inrange(r1systo,140,300) | inrange(r1diasto,90,150)) & !mi(r1systo) & !mi(r1diasto)

label variable r1mhibp "r1mhibp:w1 has measured hypertension"

label values r1mhibp yesnobase

***stage measured hibp***

gen r1smhibp = .

replace r1smhibp = 1 if inrange(r1systo,0,129.99) & inrange(r1diasto,0,84.99)

replace r1smhibp = 2 if (inrange(r1systo,130,139.99) | inrange(r1diasto,85,89.99)) & !mi(r1systo) & !mi(r1diasto)

replace r1smhibp = 3 if (inrange(r1systo,140,159.99) | inrange(r1diasto,90,99.99)) & !mi(r1systo) & !mi(r1diasto)

replace r1smhibp = 4 if (inrange(r1systo,160,179.99) | inrange(r1diasto,100,109.99)) & !mi(r1systo) & !mi(r1diasto)

replace r1smhibp = 5 if (inrange(r1systo,180,300) | inrange(r1diasto,110,150)) & !mi(r1systo) & !mi(r1diasto)

label variable r1smhibp "r1smhibp:w1 stage measured hypertension"

label values r1smhibp stage

***total hibp***

gen r1tothibp = .

replace r1tothibp = 0 if r1hibpe==0 & r1mhibp==0

replace r1tothibp = 1 if (r1hibpe==1 | r1mhibp==1) & !mi(r1hibpe) & !mi(r1mhibp)

label variable r1tothibp "r1tothibp:w1 has total hypertension"

label values r1tothibp yesnobase

***diagnosed among hypertensives***

gen r1diaghibp = .

replace r1diaghibp = 0 if r1tothibp==1

replace r1diaghibp = 1 if r1hibpe==1

label variable r1diaghibp "r1diaghibp:w1 diagnosed if hypertensive"

label values r1diaghibp yesnobase

***taking medication among hypertensives***

gen r1hibprx = .

replace r1hibprx = 0 if r1tothibp==1

replace r1hibprx = 1 if ht002c==1

label variable r1hibprx "r1hibprx:w1 on medication if hypertensive"

label values r1hibprx yesnobase

***controlling diet among hypertensives***

gen r1hibpdiet = .

replace r1hibpdiet = 0 if inlist(r1tothibp,0,1)

replace r1hibpdiet = 1 if ht002d==1

label variable r1hibpdiet "r1hibpdiet:w1 controlling diet if hypertensive"

label values r1hibpdiet yesnobase

***taking medication or controlling diet among hypertensives***

gen r1hibprxdt = .

replace r1hibprxdt = 0 if r1hibprx==0 | r1hibpdiet==0

replace r1hibprxdt = 1 if r1hibprx==1 | r1hibpdiet==1

label variable r1hibprxdt "r1hibprxdt:w1 on meds or diet if hypertensive"

label values r1hibprxdt yesnobase

***bp controlled among hypertensives***

gen r1hibpcon = .

replace r1hibpcon = 0 if r1tothibp==1

replace r1hibpcon = 1 if r1mhibp==0 & r1tothibp==1

label variable r1hibpcon "r1hibpcon:w1 bp controlled if hypertensive"

label values r1hibpcon yesnobase

***hypertensive sample***

gen samplehyp1 = 0

replace samplehyp1 = 1 if !mi(r1agegrp5) & !mi(ragender) & !mi(r1educgrp) & !mi(h1contert) & ///

!mi(hh1rural) & !mi(r1personalhi) & !mi(r1hibprx) & ///

!mi(hh1state) & !mi(r1phcchf) & !mi(r1prichf) & r1tothibp==1

***whole non-missing sample***

gen samplewhl1 = 0

replace samplewhl1 = 1 if !mi(r1agegrp5) & !mi(ragender) & !mi(r1educgrp) & !mi(h1contert) & ///

!mi(hh1rural) & !mi(r1personalhi) & !mi(r1hibprx) & ///

!mi(hh1state) & !mi(r1phcchf) & !mi(r1prichf) & !mi(r1tothibp)

***save dataset***

save "baseline", replace
